# Hibiscus attenuates renovascular hypertension–induced aortic remodeling dose dependently: the oxidative stress role and Ang II/cyclophilin A/ERK1/2 signaling

**DOI:** 10.3389/fphys.2023.1116705

**Published:** 2023-06-21

**Authors:** Asmaa Mohammed ShamsEldeen, Ahmed Fawzy, Hend Ashour, Marwa Abdel-Rahman, Hend Elsayed Nasr, Lina Abdelhady Mohammed, Noha Samir Abdel Latif, Amr M. Mahrous, Shereen Abdelfattah

**Affiliations:** ^1^ Department of Physiology, Faculty of Medicine, Cairo University, Giza, Egypt; ^2^ Department of Physiology, Faculty of Medicine, King Khalid University, Abha, Saudi Arabia; ^3^ Department of Medical Biochemistry and Molecular Biology, Benha University, Benha, Egypt; ^4^ Department of Pharmacology, Faculty of Medicine, Cairo University, Giza, Egypt; ^5^ Department of Pharmacognosy, Faculty of Pharmacy, El Saleheya El Gadida University, Al Sharquia, Egypt; ^6^ Department of Anatomy and Embryology Faculty of Medicine, Cairo University, Giza, Egypt

**Keywords:** hibiscus, MALAT1, aortic response, 2K1C, cyclophilin A, ERK1/2

## Abstract

**Introduction:** The high levels of angiotensin II (Ang II) can modify the vascular tone, enhance vascular smooth muscle cells (VSMCs) proliferation and hypertrophy and increase the inflammatory cellular infiltration into the vessel wall. The old herbal nonpharmacological agent, Hibiscus (HS) sabdariffa L has multiple cardioprotective impacts; thus, we investigated the role of HS extract in amelioration of renovascular hypertension (RVH)-induced aortic remodeling.

**Materials and methods:** Thirty-five rats (7/group) were randomly allocated into 5 groups; group: I: Control-sham group, and RVH groups; II, III, IV, and V. The rats in RVH groups were subjected to the modified Goldblatt two-kidneys, one clip (2K1C) for induction of hypertension. In group: II, the rats were left untreated whereas in group III, IV, and V: RVH-rats were treated for 6 weeks with low dose hibiscus (LDH), medium dose hibiscus (MDH), and high dose hibiscus (HDH) respectively.

**Results:** We found that the augmented pro-contractile response of the aortic rings was ameliorated secondary to the *in-vivo* treatment with HS dose dependently. The cyclophilin A (CyPA) protein levels positively correlated with the vascular adhesion molecule-1 (VCAM-1) and ERK1/2, which, in turn, contribute to the reactive oxygen species (ROS) production. Daily HS intake modified aortic renovation by enhancing the antioxidant capacity, restraining hypertrophy and fibrosis, downregulation of the metastasis associated lung adenocarcinoma transcript (MALAT1), and cyclophilin A (CyPA)/ERK1/2 levels.

**Discussion:** Adding to the multiple beneficial effects, HS aqueous extract was able to inhibit vascular smooth muscle cell proliferation induced by 2K1C model. Thus, adding more privilege for the utilization of the traditional herbal extracts to attenuate RVH-induced aortopathy.

## 1 Introduction

Controlling blood pressure is an imperative goal for tumbling the associated structural and functional impediments that could disturb different organs. Formerly, published human and animal work have reported vascular alterations subsequent to hypertension (Rodrigo et al., 2013; Schiffrin, 2015). Large arteries, principally aorta, undergo hypertrophic changes such as phenotypic interchanging in smooth muscle cells, augmented depositing of collagen through a decrement in the ratio of elastic/collagen, and malfunctioning in elastic production (Basu et al., 2010). Moreover, vascular stiffness might hasten blood pressure upsurge. Amplified stiffness and reduced aortic diameter in middle-aged hypertensive patients and the presence of disease-linked modifications (e.g., atherosclerosis) may boost specific impedance, thereby increasing pulse pressure (Gesche et al., 2012).

Former pioneering studies of Goldblatt et al. (1934) documented the development of chronic renovascular hypertension (RVH) following unilateral renal artery ligation. The developed RVH occurred secondary to both renal ischemia and the activation of the renin–angiotensin system (RAS) (Andersson et al., 2017). High levels of angiotensin II (Ang II) could alter vascular tone, stimulate vascular smooth muscle cell (VSMC) proliferation and hypertrophy, enhance the production of pro-inflammatory cytokines, and influence macrophage infiltration into vascular walls (Pathak et al., 2016).

In rat aortic VSMCs, Ang II-induced hypertrophy was reliant on reactive oxygen species (ROS) overproduction (Yin et al., 2020), whereas cyclophilin A (CyPA), the chaperone protein expressed in VSMCs, could enhance their migration and proliferation, while the deficiency of this protein blocked Ang II-mediated vascular inflammation and decreased aortic inflammatory cell recruitment (Satoh et al., 2009). In the *in vitro* study conducted by Jin et al. (2000), CyPA secreted in response to oxidative stress could enhance ERK1/2 expression in VSMCs.

Cellular behaviors of VSMCs could be epigenetically regulated by long non-coding RNAs (lncRNAs), one of which is metastasis-associated lung adenocarcinoma transcript 1 (MALAT1) (Cheng et al., 2019). MALAT1 overexpression was observed in the aortic tissues of spontaneous hypertensive rats and was linked to VSMC proliferation and fibronectin and collagen I deposition (Li et al., 2019), whereas its downregulation could alleviate hypertension-mediated vascular remodeling in rats (Xue et al., 2019).

*Hibiscus sabdariffa* L, which is commonly known to be used in cold beverages or hot drinks, is assumed to have an antihypertensive influence (Mensah and Golomeke, 2015). Alarcón-Alonso et al. (2012) reported on the diuretic impact that HS has and the subsequent decrease in serum Na^+^, K^+^, and phosphate in humans. Preceding studies have reported the antioxidant effects of HS (Chou et al., 2019). In Marfan syndrome, infusion of HS could not only mend oxidative stress by triggering vascular superoxide dismutase (SOD) and glutathione peroxidase (GPx) production but also increase the total antioxidant capacity (TAC) (Soto et al., 2016).

Taken together, we hypothesized that in response to oxidative stress that is associated with sustained hypertension, the expression of CyPA may be increased in aortic tissues, leading eventually to increased ERK1/2 protein levels. We investigated the vascular protection and antioxidant properties exerted by HS aqueous extract in a rat model of RVH and conducted *in vitro* work to study the potential response of vascular smooth muscle tissue (isolated aorta) to phenylephrine.

## 2 Materials and methods

### 2.1 Study design

The experimental design was considered and approved by the local scientific committee of the Anatomy Department, Faculty of Medicine, Cairo University, and the experimental procedures were carried out in physiology, pharmacology, biochemistry, and molecular biology, while tissue examination was conducted in the Anatomy and Embryology departments. The animals were purchased and housed in the animal facility of the Cairo University. Thirty-five male Wistar rats aged approximately 8 weeks and weighting 180–200 g were maintained in the animal facility, following the Guide for the Care and Use of Laboratory Animals, and their body weights measured regularly using the CLASCO SF-400 body weighting scale (Supplementary Figure S1). After acclimatizing to ordinary environmental living conditions, the animals were kept in wire-mesh cages (three rats per cage) and given free access to food and water before starting any experimental procedure.

### 2.2 Animal grouping

The rats were fasted overnight before the experiments. The experimental animal protocols were done according to the ARRIVE guidelines and the Guide for the Care and Use of Laboratory Animals of the Cairo University, approval no. CU/III/F/47/22. The animals included in this study were randomly divided into the following five main groups (seven mice in each group):

Group I—(sham-operated) control group (n = 7): after determination of the third stage of anesthesia, a 2-cm-long incision was made 0.5 cm away from the vertebral column, just below the ribs on the left side. The left renal artery was exposed, and no further surgical manipulations were done (Alam et al., 2015).

Group II—RVH group (n = 7): this group underwent the same procedure as the sham-operated one, but with unilateral clipping of the renal artery for inducing the RVH model (Alam et al., 2015).

The other groups were supplemented with HS aqueous extract as described in Section 2.5.1.

Group III—RVH-LDH group (n = 7): rats in this group were given a low dose hibiscus (LDH) in drinking water at a concentration of 5 mg/mL.

Group IV—RVH-MDH group (n = 7): rats in this group were given a medium dose of HS (MDH) in drinking water at a concentration of 10 mg/ml.

Group V—RVH-HDH group (n = 7): rats in this group were given high dose of HS (HDH) in drinking water at a concentration of 20 mg/mL.

The rats were supplemented with HS aqueous extract after confirmation of hypertension (2 weeks postoperative period) with continued supplements (for 6 weeks) till the end of the study (Ribeiro et al., 2013).

### 2.3 Induction of renovascular hypertension using modified Goldblatt two-kidney, one-clip method for clipping of left renal artery

Hypertension was established using the modified Goldblatt method [two-kidney, one clip (2K1C)]. The rats were anesthetized by a single intraperitoneal injection of cocktail (ketamine–xylazine) solution that was given at a dose of 0.15 mL/100 g body weight. The prepared cocktail solution was formed of ketamine that was given at a dose of 100 mg/kg and xylazine at a dose of 10 mg/kg (Flecknell, 2009). After shaving the hair and sterilizing the skin in the left side using a topical antiseptic, a 2-cm-long incision was made just below the ribs, 0.5 cm away from the vertebral column. The left kidney was exposed and the renal artery was identified; it was dissected from the surrounding connective tissue (Alam et al., 2015), and a silver clip was placed with a 300-μm-wide gap around it. After this, the muscle layer was closed by continuous sutures using 4-0 silk, and finally, the skin was closed with interrupted sutures using 3-0 silk.

### 2.4 Postoperative care of animals

Each rat was injected daily with penicillin G (100,000 units IM) for three successive days (Douglas et al., 1976) and a topical antibiotic applied twice daily.

### 2.5 Hibiscus preparation and high-performance liquid chromatography analysis of flavonoids and polyphenolic compounds

#### 2.5.1 Hibiscus preparation and administration

*Hibiscus sabdariffa* L. was taken from the experimental station for medicinal plants at the Faculty of Agriculture, Cairo University, Egypt. The flowers were harvested and the calyces were separated, then they were dried (a voucher specimen 08-06-2018). The dried calyces were used to produce the aqueous extract. Three different concentrations were prepared by placing 5, 10, and 20 g of dried HS in 995-, 990-, and 980-mL volume bottles of distilled water, respectively, to produce the low-, medium-, and high-dose HS concentrations of 5 mg/mL, 10 mg/mL, and 20 mg/mL, respectively. The bottles were shaken for 5 minutes and left overnight at room temperature. After 24 h, the produced water extracts were given to the rats as the only source for drinking (Inuwa et al., 2012).

#### 2.5.2 HPLC standardization of hibiscus crude powder

The Waters Alliance 2690 HPLC system equipped with the Waters 996 Photodiode Array Detector and an Automatic Sample Injection System, In-Line Degasser AF, and ultraviolet detector set at 280 nm for flavonoids and phenolic acid, and HP pump were used for the standardization of the prepared extract. About 0.5 gm of the HS sample was weighed into a 100-mL Erlenmeyer flask and then dispersed in 100 mL of distilled water to produce the aqueous extract. Before quantification by using HPLC, the sample was filtered through a 0.2-µm membrane filter. The separation and determination of flavonoids were performed by reverse phase HPLC (RP-HPLC) diode array detection (DAD), and the wave length used for identification and qualification of flavonoids with the diode array was 280 nm, using a Kromasil C18 column (5 µm, 4.6 × 250 mm; PerkinElmer, Inc., United States) with a guard column. The temperature of the column oven was set at 30°C.

A gradient elution system was employed for the flavonoids and phenolic acid. The flow rate of the mobile phase was 1 mL/min, and the injection volume was 10 µL for the standards and sample extracts. Detection was carried out at 280 nm. Serial dilutions (10, 20, 30, 40, 50, and 60 μg/mL) of rutin (99% purity), gallic acid (99% purity), and quercetin (99% purity) were prepared from the stock solution having 1 mg/mL concentration. All flavonoids and phenolic acid were quantified using an external standard method. Quantification was achieved using DAD based on peak area. The results of the two types of flavonoids and one phenolic acid in HS were expressed as the mean of three determinations.

The aqueous extract of *H. sabdariffa* was co-injected with known concentrations of gallic acid, rutin, and quercetin as the internal standard at 280 nm. The characteristic peaks 1, 2, and 3, at the retention times of 10.127, 45.691, and 55.005 min, respectively, correspond to previously reported phenolic acids and flavonoids (Kamal et al., 2017)*.*

### 2.6 Blood pressure measurements

The blood pressure (BP) of the animals was indirectly measured with the non-invasive blood pressure monitor (LE 5001 Pressure Meter, Letica Scientific Instruments, Spain) from the tail of the conscious rats by using the tail-cuff technique.

In the tail-cuff technique, the animals were warmed for 30 min at 28°C in a thermostatically controlled heating cabinet (Ugo Basile, Italy) for better detection of the pulse in the tail artery. The tail was passed through a miniaturized cuff and a tail-cuff sensor that was connected to an amplifier (LE 5001 Pressure Meter, Letica Scientific Instruments, Spain) (Irvine et al., 1997).

The amplified pulse was recorded during automatic inflation and deflation of the cuff. The systolic blood pressure was defined as the cuff inflation pressure at which the waveform becomes indistinguishable from the baseline noise. The average of at least three measurements was taken on each occasion. The tail cuff is a common and convenient non-invasive method to measure the systolic pressure in rats. The cuff is attached to a tail cuff sphygmomanometer, and the BP is recorded on a chart (Nagura et al., 1996). All the rats were subjected to arterial blood pressure assessment 3 times: the first was baseline assessment, and the mean values of the systolic arterial blood pressure (SABP) showed no significance between the groups; then again for hypertension confirmation (2 weeks postoperative period), and the last recording at the end of the study.

### 2.7 Sample collection and animal sacrifice

1) At the planned time, the animals were subjected to arterial blood pressure assessments. intra-The method of blood collection via intra-orbital route from the retro-orbital plexus using heparinized capillary tubes. The collected samples were centrifuged at 3,000 rpm for 15 min at 4°C, and the obtained sera were used for the estimation of creatinine, levels of nitric oxide (NO), angiotensin II, vascular cell adhesion molecule (VCAM-1), nuclear factor kappa beta (NFк-B), tumor necrosis factor alpha (TNF-α), and interleukin (IL-10). The aortic levels of malondialdehyde (MDA), 8-hydroxy-2′-deoxyguanosine (8-OHdG), the total antioxidant capacity (TAC), and superoxide dismutase (SOD) were also measured. Furthermore, the aortic lncRNA metastasis-associated lung adenocarcinoma transcript 1 (lncRNA-MALAT1) gene expression and cyclophilin A, and extracellular receptor kinase (ERK1/2) protein levels were estimated.
2) The animals were euthanized, and the thoracic aorta was quickly removed and cleaned of the connective tissue. The aortic tissues were harvested and subjected to *in vitro* recording of the vascular reactivity in response to different concentrations of phenylephrine; furthermore, the biochemical and histological examinations were carried out.

### 2.8 *In vitro* assessment of aortic response

The aortic tissues were dissected from the animals treated *in vivo* with different concentrations of HS. The assessment of isolated aortic rings response to different doses of phenylephrine was done *in vitro*. The aortic tissues were cut into rings of approximately of 3–4 mm in diameter and transferred to organ chambers filled with 10 mL of freshly prepared Krebs–Henseleit solution [NaCl (118 mM), KCl (4.7 mM), CaCl_2_ (2.5 mM), MgSO_4_ (1.2 mM), NaHCO_3_ (25 mM), KH_2_PO_4_ (1.2 mM), and glucose (10 mM)] maintained at 37°C, at a pH of 7.4, and gassed with 95% O_2_ and 5% CO_2_. Then, the rings were mounted between two hooks attached to an isometric force transducer connected to a data acquisition system (PowerLab/8SP, ADInstruments) for the continuous recording of tension of 1 g for 1 h (Csanyi et al., 2007). When the aortic response was done, each aortic ring was subjected *in vitro* to the concentrations of phenylephrine (10 μg, 20 μg, and 40 µg) in the organ bath.

### 2.9 Use of paraffin sections

#### 2.9.1 Light microscopic study

1. Hematoxylin and eosin stain: to investigate the cytoplasmic and nuclear alterations in the cells.
2. Masson’s trichrome stain: to examine the distribution of collagen fibers, paraffin units were dewaxed, rehydrated ,then stained in acid fuchsin solution (5 min), dipped in distilled water, placed in phosphomolybdic acid solution (3 min), splashed in distilled water, stained with the methyl blue (2–5 min), bathed in distilled water, and preserved in acetic acid for 2 min. To complete, they were dehydrated in alcohol, cleared in xylol, and fixed in Canada balsam. The collagen fibers appeared blue.
3. Orcein stain: to observe the elastic fibers, the aortic sections were deparaffinized and hydrated, then potassium permanganate solution was added (10 min) and the sections were rinsed in water, treated with 5% oxalic acid until colorless, rewashed in tap water, rehydrated in distilled water, treated with 0.5% Periodic acid (5 min), and washed and rinsed again in distilled water. The sections were then treated with orcein solution and microwaved in low power for 30–45 s, and then left to stand for 30 min. When observed under the microscope, it was found that the elastic fibers appeared dark brown, while the background appeared light brown.

#### 2.9.2 Immunohistochemistry

Three percent hydrogen peroxide solution was used to block the endogenous peroxidase action. For recovering tissue antigen, a microwave was used. Antigen retrieval was accomplished by heating the sections in 10 mM sodium citrate buffer, in a water bath (95°C–100°C; 30 min). The units were soaked and positioned in PBS (5 min) subsequently; then, ordinary goat serum was supplemented (30 min) at room temperature. Afterward, the sections were incubated with the primary α-SMA antibody (ab7817, diluted 1:100), primary rabbit polyclonal TNF-α antibody (ab66579, diluted 1:500), and anti-eNOS antibody (ab5589, diluted 1:100). The sections were subsequently incubated with goat anti-rabbit IgG H&L (HRP; ab205718) for 20 min at 37°C. DAB (3,3′-diaminobenzidine) was used as the chromogen, and all the immunohistochemical sections were visualized using DAB (Sigma-Aldrich) and observed under a microscope to witness the progression of color. Following the washing with distilled water, the sections were counterstained with hematoxylin, permeabilized with xylene, mounted with resin, and treated with decreasing ethanol series. All the antibodies were purchased from Abcam, Cambridge, UK. Negative control sections were processed using the same abovementioned procedure, except that the primary antibody was replaced by non-immune mouse serum immune reaction.

#### 2.9.3 Image analysis and morphometric measurements

The thickness of the tunica media, percentage of area occupied by collagen and elastic fibers, and immune expressions of α-SMA, TNF-α, and e-NOS were obtained from the Leica LAS V3.8 image analyzer computer system (Leica, Switzerland). The measurements were self-determined by a blinded viewer. The data were acquired from 10 non-overlapping microscopic fields randomly occupied on each slide and inspected using the standard measuring frame at a magnification of ×400. Within the measuring frame, the percentage of area was identified and screened by using a binary color.

### 2.10 Serum samples for measuring biochemical parameters

#### 2.10.1 Estimating serum creatinine level

The serum creatinine level was measured by the colorimetric method using QuantiChrom™ assay kit, and all steps were performed according to the Jung and Jaffe methods (DIUR-500 and DICT-500).

#### 2.10.2 Estimating serum levels of nitric oxide, angiotensin II, and vascular cell adhesion molecule-1

The NO level was determined by estimating the total nitrite/nitrate level using the Nitric Oxide Assay Kit (Colorimetric) (Catalog # ab65328, Abcam). The concentration of each sample was calculated, and the results are expressed as nmol/mL.

For measuring the level of Ang II, the Rat Enzyme-Linked Immunosorbent Assay (ELISA) Kits (RAB0010-1KT, Sigma-Aldrich, St. Louis, MO, United States) were purchased, and the concentration of plasma Ang II was determined according to the assay kit’s instructions.

For estimating the level of vascular cell adhesion molecule-1 (VCAM-1), the Rat VCAM-1 ELISA Kit (Catalog # MBS2502676, MyBioSource) was used, and all the steps were done according to the sandwich ELISA principle.

#### 2.10.3 Estimating serum nuclear factor kappa beta, tumor necrosis factor alpha, and interleukin-10

The serum levels of the nuclear factor kappa beta (NF-κB) by using the Rat Nuclear Factor Kappa B (NF-kB) ELISA Kit (Catalog # MBS453975, MyBioSource), tumor necrosis factor alpha (TNF-α) by using the TNF-α Rat ELISA Kit (Catalog # KRC3012, Invitrogen), and interleukin (IL-10) by using the Rat IL-10 ELISA Kit (Catalog # ERA23RB, Invitrogen) were measured according to the manufacturer’s recommendations.

#### 2.10.4 Measuring pro-oxidant (MDA and 8-OHdG) and antioxidant (TAC and SOD) markers in aortic tissues

The MDA levels were determined using the thiobarbituric acid reactive substance (TBARS) according to the procedure of Albro et al. (1986). Approximately 2.5 mL of 10% thiobarbituric acid (TBA) was added to 0.5 mL of the tissue homogenate, and then the mixture was heated for 30 min in a boiling water bath. The formed chromogen was extracted using 4 mL of N-butyl alcohol, and the absorbance was determined at the wavelength of 532 nm.

The concentration of 8-hydroxy-2′-deoxyguanosine (8-OHdG) was determined by utilizing the Rat 8-OHdG ELISA Kit (Catalog # ab285302, Abcam). Approximately 100 μL of the HRP-conjugate reagent was added to each diluted sample in each well and incubated for 60 min at 37°C. The plate sealer was removed, and the plates were refilled with the washing solution. After resting for 30 s, the washing procedure was repeated 5 times following the same steps. The prepared chromogen solutions were added to each well and incubated at 37°C for 15 min. The stop solution was added to each well, and the absorbance was determined at 450 nm.

#### 2.10.5 Total antioxidant capacity

Using the Rat Total antioxidant status (TAC) ELISA Kit (Catalog # MBS1600693, MyBioSource), the total antioxidant status was measured, and all the procedures were performed following the manufacturer’s instructions. The sandwich ELISA kit was utilized for the accurate quantitative detection of the total antioxidant status in the aortic tissue homogenate, and the reaction was terminated by the addition of the acidic stop solution and absorbance was measured at 450 nm.

#### 2.10.6 Superoxide dismutase

The SOD activity was estimated as described previously (Alam et al., 2015); the aliquots of tissue homogenates were added to epinephrine with phosphate buffer saline (PBS), and at 15 s intervals, the reaction was observed and the absorbance change was noted for a minute at 480 nm. In this experiment, the method is based on the ability of SOD to inhibit the reduction of nitroblue tetrazolium (NBT) by superoxide anions generated by pyrogallol autoxidation. A SOD unit is defined as the amount of enzyme that causes 50% inhibition of the autoxidation of pyrogallol under assay conditions.

#### 2.10.7 Quantitative real-time PCR analyses for MALAT1 expression in aortic tissues

After the homogenization of the aortic samples, total RNA containing miRNA was determined utilizing TRIzol^®^ (Invitrogen; Thermo Fisher Scientific Inc.) and extracted. Then, the RNeasy purification reagent (obtained from Qiagen, Valencia, CA) was utilized for RNA isolation, and the purity of the extracted RNA was detected by using a spectrophotometer. Reverse transcription of the extracted RNA to a complementary DNA was performed using the reverse transcription reaction (SuperScript II; Gibco Life Technologies, Grand Island, NY). Real-time PCR amplification and analysis were performed by using SYBR Green PCR core reagents (Applied Biosystems, Foster City, CA, United States) and 0.2 μM of specific primers for rat MALAT1. The expression of β-actin messenger RNA was used as an internal control housekeeping gene in all the samples. The primer sequences of the studied genes are as follows: MALAT1 Forward: 5′-AAA​GCA​AGG​TCT​CCC​CAC​A-3′, Reverse: 5′-TGT​GGG​GAG​ACC​TTG​CTT​T-3′, accession no. LC685074.2, and β-actin (ACTB) Forward: 5′-AGC​CAT​GTA​CGT​AGC​CAT-3′, Reverse: 5′-ATG​GCT​ACG​TAC​ATG​GCT-3′, accession no. NM_031144.2.

#### 2.10.8 Western blot detection of cyclophilin A and ERK1/2 in aortic tissues

After tissue homogenization, proteins were extracted, and their concentrations were determined in each sample using the Bradford protein assay. The same amounts of protein (30 µg) were fractionated using 10% sodium dodecyl sulfate polyacrylamide gel electrophoresis, and the steps were done using a Bio-Rad Mini-PROTEAN II system (Hoefer Inc., Holliston, MA). In polyvinylidene difluoride membranes, proteins samples were washed with PBS and blocked with 5% (wt/vol) skimmed milk powder in PBS. Then, blots were obtained using antibodies for cyclophilin A (#2175, 1:1000 dilution, 18 KDa), ERK1/2 (#9102, 1:1000 dilution, 42,44 KDa), and β-actin (#4970,1:1000 dilution, 45 KDa); the antibodies were obtained from Cell Signaling Technology, Inc., Danvers, MA, United States. All samples were incubated with the primary antibodies overnight at a pH of 7.6, in 4°C with continuous gentle shaking. The membranes were washed in Tween-20 and incubated with the secondary antibody (1:10,000 dilution) that was obtained from Santa Cruz Biochemicals, at 37°C. The band intensities were estimated and analyzed with the ChemiDoc™ Imaging System. The data were analyzed relative to the levels of housekeeping β-actin protein level.

### 2.11 Statistical methods

Data were collected and entered using the Statistical Package for the Social Sciences (SPSS) version 20 (IBM Corp., Armonk, NY, United States). The tests for normality; Kolmogorov–Smirnov and Shapiro–Wilk revealed normally distributed data (Supplementary Material, tests for normality). Data are summarized and presented as mean and standard deviation. Comparisons between groups were done using the analysis of variance (ANOVA) with Tukey's *post hoc* multiple comparisons test. *p*-values less than 0.05 were considered statistically significant.

## 3 Results

### 3.1 HPLC results of flavonoids and polyphenolic compounds present in hibiscus (µg/100 g)

The co-injected aqueous extract of *H. sabdariffa* with gallic acid, rutin, and quercetin as the internal standard at 280 nm had characteristic peaks 1, 2, and 3 at the retention times 10.127, 45.691, and 55.005 min, respectively. The assessed *H. sabdariffa* L. that was used in the experiment was effective and was subjected to further clinical trials (Table 1; Supplementary Figure S2).

**TABLE 1:** HPLC results of flavonoids and polyphenolic compounds present in Hibiscus (µg/100 g).

No.	Test item	Retention time	Concentration (mg of std/gm of extract)
1	Gallic acid	10.127	0.107 ± 0.004
2	Rutin	45.691	3.09 ± 0.03
3	Quercetin	55.005	15.6 ± 0.010

### 3.2 Influence of hibiscus on arterial blood pressure

The systolic blood pressure levels were statistically evaluated 3 times. The baseline data reflected no statistical difference between the groups. Two weeks after surgery, the systolic blood pressure was significantly higher in all rats exposed to 2K1C operation than in the control group, indicating successful induction of hypertension. At the end of the study, the mean values of systolic blood pressure were significantly increased in all RVH groups when compared to that in the control group. However, HS efficacy in decreasing the systolic blood pressure was clearly detected in all treated groups when compared with the RVH group, and the maximum control over the arterial blood pressure was recorded in the RVH-HDH group when compared with the other treated groups (Table 2).

**TABLE 2:** Changes in systolic blood pressure in the different experimental groups. LDH, MDH, and HDH are equivalent to concentrations 5 mg/mL, 10 mg/mL, and 20 mg/mL, respectively. Data are presented as mean ± SD. *p* ˂ 0.05 is considered significant. Statistical significance is denoted by “β” when compared with control 2 weeks after induction of RVH, * when compared with control at the end of the study, # when compared with RVH, $ when compared with RVH-LDH, ^ when compared with RVH-MDH, and % when compared with RVH-HDH.

Group		Systolic blood pressure (mmHg)		
		Baseline	Two weeks	End of study
Control	Mean ± SD	116 ± 3.23	114.6 ± 2.37	114 ± 2.31
RVH	Mean ± SD	115 ± 3	217.7 ± 6.18 β	227 ± 6.24 *$%
RVH-LDH	Mean ± SD	117 ± 1.63	216 ± 5.77 β	180.14 ± 5.34 *#%
RVH-MDH	Mean ± SD	117.1 ± 1.77	216.1 ± 5.76 β	167.29 ± 8.2 *#$%
RVH-HDH	Mean ± SD	116.6 ± 1.40	216.86 ± 2.67 β	153.71 ± 6.40 *#$^

### 3.3 Attenuation of phenylephrine-induced aortic tension

The non-cumulative aortic response to the different doses of phenylephrine (10, 20, and 40 µg) was recorded. In this regard, our data showed significant increase in developed aortic tension in the RVH group in response to all doses of phenylephrine (0.55 ± 0.03, 0.75 ± 0.03, and 1.43 ± 0.03) when compared to the responses in the control group (0.13 ± 0.01, 0.23 ± 0.01, and 0.34 ± 0.028).

The result of aortic contractility in response to phenylephrine was significantly decreased in the RVH-HDH group (0.14 ± 0.024, 0.22 ± 0.026, and 0.37 ± 0.034) when compared with both the RVH-MDH (0.17 ± 0.01, 0.32 ± 0.04, and 0.46 ± 0.03) and RVH-LDH (0.26 ± 0.03, 0.46 ± 0.05, and 0.48 ± 0.025) groups.

**FIGURE 1:**
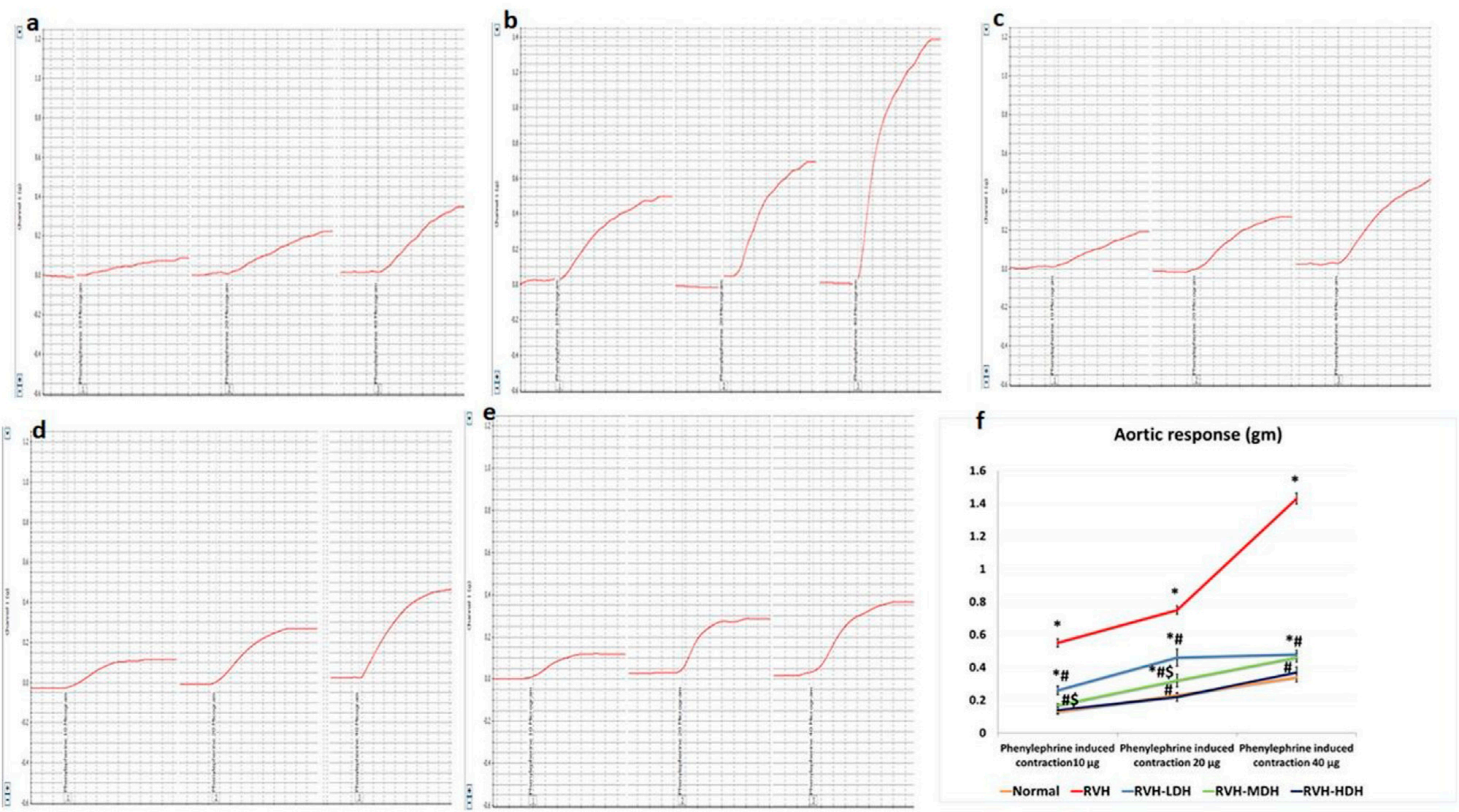
Recorded PowerLab chart showing aortic vascular contractile response to different doses of phenylephrine (PE: 10, 20, and 40 µg/15 mL bath solution) in all studied groups. **(A)** Control group. **(B)** Group II (RVH). **(C)** Group III (RVH-LDH). **(D)** Group IV (RVH-MDH). **(E)** Group V (RVH-HDH). **(F)** Column curve presents aortic vascular response to different doses of phenylephrine in all studied groups. HS could dose dependently attenuate the response of aorta to PE. In addition, normalization of vascular contractility was observed in the RVH-HDH group when compared with the control group. RVH: renovascular hypertension, LDH: low dose hibiscus, MDH: medium dose of hibiscus, and HDH: high dose of hibiscus. LDH, MDH, and HDH are equivalent to concentrations of 5 mg/mL, 10 mg/mL, and 20 mg/mL, respectively. Statistical significance is denoted by *when compared with the control group, #when compared with the RVH group, $when compared with the LDH, and ^when compared with the MDH group. Analysis was done using ANOVA, followed by Tukey’s *post hoc* test (n = 7); significance was considered when *p* ˂ 0.05.

In addition, normalization of vascular contractility was observed in the RVH-HDH group (0.14 ± 0.024, 0.22 ± 0.026, and 0.37 ± 0.03) when compared with the control group (0.13 ± 0.01, 0.23 ± 0.01, and 0.34 ± 0.028) (Figure 1).

### 3.4 Light microscopic examination; general configuration of aorta as shown by H&E staining: amount of collagen and elastic fibers

**FIGURE 2:**
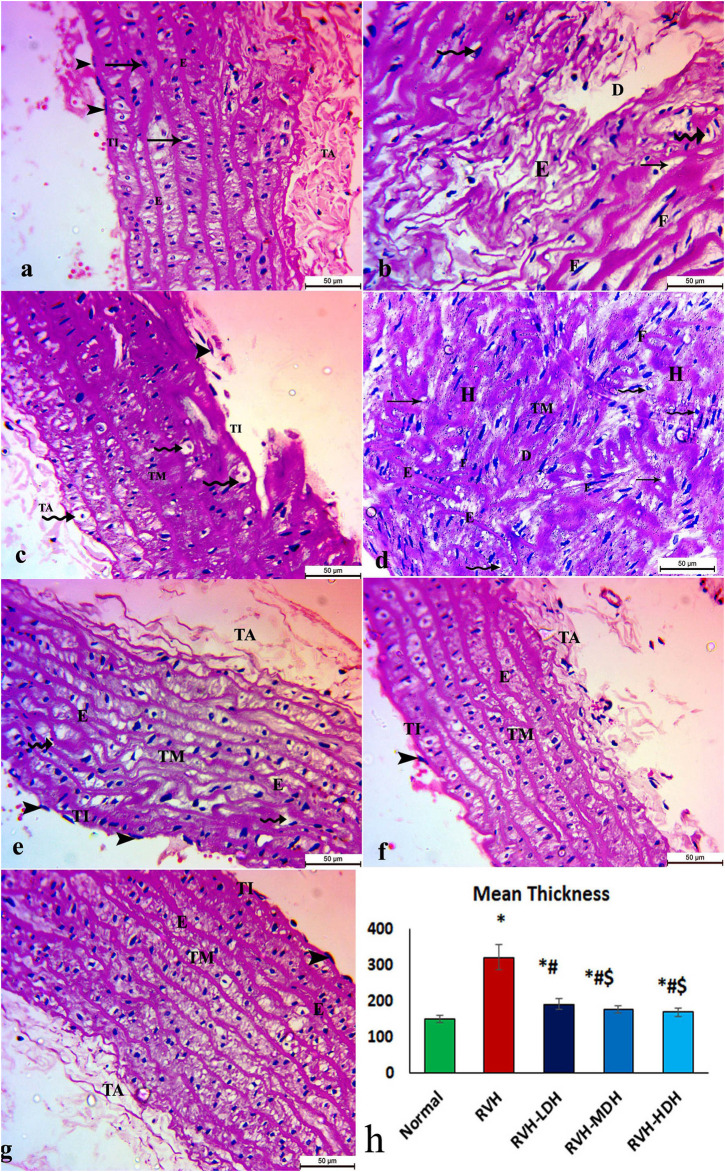
Micrograph of an aortic section of adult male albino rat of **(A)** control groups: aortic wall—tunica intima (TI) is thin and possesses endothelial cells (arrow heads); tunica media (TM) encompasses elastic fibers **(E)**, smooth muscle (arrows), and collagen tissue; and finally, tunica adventitia (TA) is the outermost component of the arterial wall. **(B,C,D)** The RVH group presents disruption in tunica intima (TI) with exfoliated cells (arrow heads). Tunica media: disturbed construction **(D)**, obvious muscular hypertrophy **(H)**, disturbed and torn elastic fibers **(E)**, and degenerated cells (vacuolated cytoplasm with pyknotic nuclei) (curved arrows). Note the existence of fibroblasts **(F)** and fat droplets (arrows). **(E,F,G)** Groups III (RVH-LDH), IV (RVH-MDH), and V (RVH-HDH) show an almost normal appearance of three aortic layers (TI, TM, and TA), but some cells still show vacuolated cytoplasm (curved arrows) in group III. **(H)** Illustrative graph for mean values of the thickness of tunica media (H&E ×400). RVH: renovascular hypertension, LDH: low dose hibiscus, MDH: medium dose of hibiscus, and HDH: high dose of hibiscus. LDH, MDH, and HDH are equivalent to concentrations of 5 mg/mL, 10 mg/mL, and 20 mg/mL, respectively. Statistical significance is denoted by * when compared with the control group, # when compared with the RVH group, $ when compared with the LDH group, and ^ when compared with the MDH group. Analysis was done using ANOVA, followed by Tukey’s *post hoc* test (n = 7); significance was considered when *p* ˂ 0.05.

The aortic sections of the control group revealed normal organization of the three layers of the aortic wall: the tunica intima, which is a thin layer and retains endothelial cells; the tunica media, which encompasses smooth muscles, elastic fibers, and collagen tissue; and lastly, the tunica adventitia, which is the outermost constituent of the arterial wall and consists of connective tissue and collagen fibers (Figure 2A). The aortic sections in the RVH group presented disruptions in the tunica intima with cellular exfoliation. The tunica media revealed disturbed construction, apparent muscular hypertrophy, and disturbed and torn elastic fibers. The cells appeared degenerate (vacuolated cytoplasm with pyknotic nuclei). The fibroblasts and fat droplets were noticeable findings in this group (Figure 2B–D). The RVH-LDH, RVH-MDH, and RVH-HDH groups showed a nearly customary appearance of the three aortic layers, nonetheless several cells still disclosed vacuolated cytoplasm in group III (RVH-LDH) (Figures 2E–G).

**FIGURE 3:**
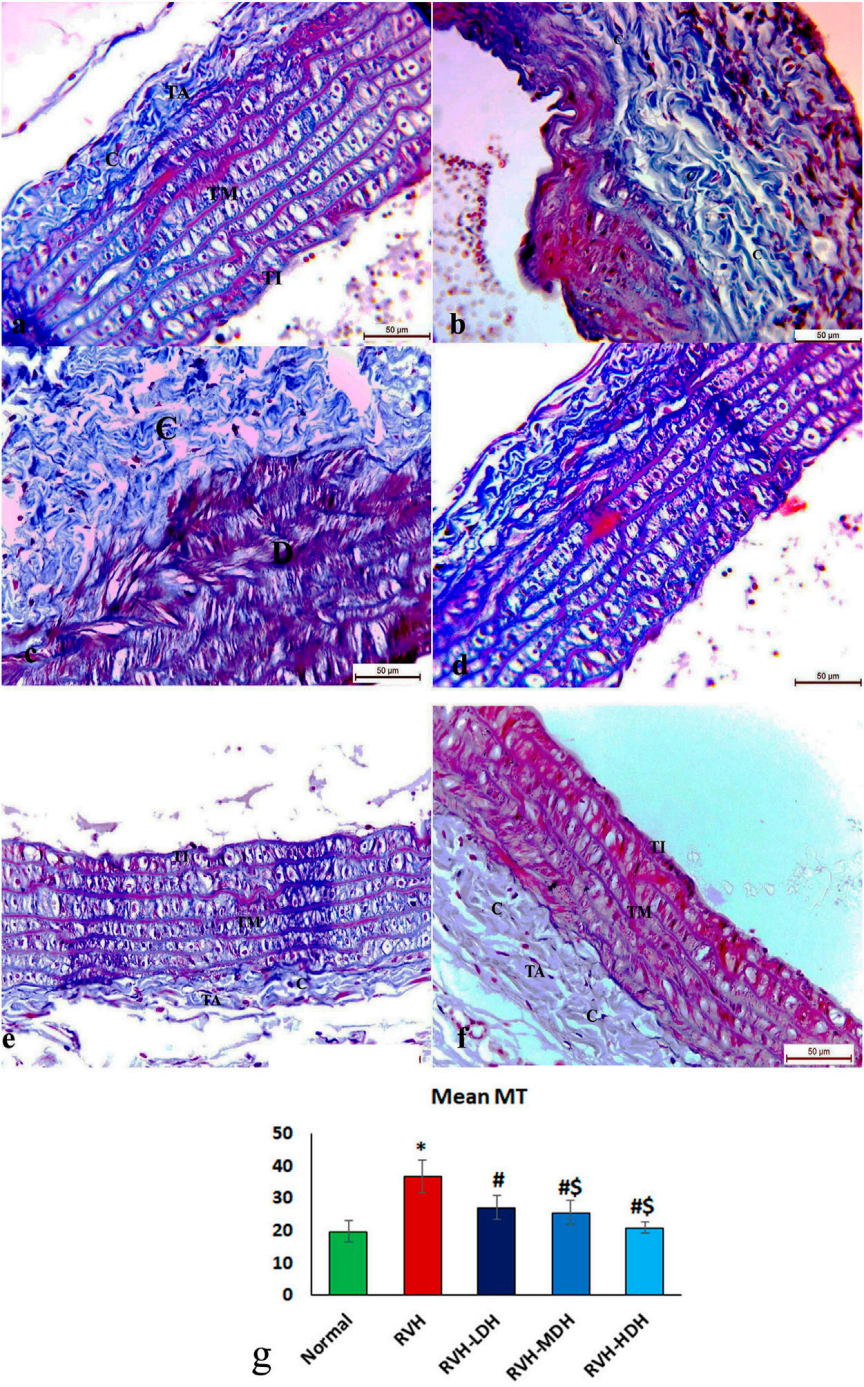
Micrograph of Masson trichrome-stained sections in the aortic section of adult male albino rats.**(A)** Group I: “C” indicates adequate dispersal of collagen fibers. **(B,C)** Group II (RVH): coarse scattering of collagen fibers. **(D,E)** Groups III (RVH-LDH) and IV (RVH-MDH): reasonable spreading of collagen fibers. **(F)** Group V (RVH-HDH): fine distribution of collagen. **(G)** Expressive graph of the mean values of area percentage of collagen fibers (Masson trichrome ×400). RVH: renovascular hypertension, LDH: low dose hibiscus, MDH: medium dose of hibiscus, and HDH: high dose of hibiscus. LDH, MDH, and HDH are equivalent to concentrations 5 mg/mL, 10 mg/mL, and 20 mg/mL, respectively. Statistical significance is denoted by * when compared with the control group, # when compared with the RVH group, $ when compared with the LDH, and ^ when compared with the MDH group. Analysis was done using ANOVA, followed by Tukey’s *post hoc* test (n = 7); significance was considered when *p* ˂ 0.05.

The control group revealed adequate dispersal of collagen fibers (Figure 3A), while the RVH group showed coarse scattering of collagen fibers (Figures 3B,C). However, the RVH-LDH and RVH-MDH groups showed reasonable spreading of collagen fibers (Figures 3D, E), and the RVH-HDH group displayed a fine distribution of collagen (Figure 3F).

**FIGURE 4:**
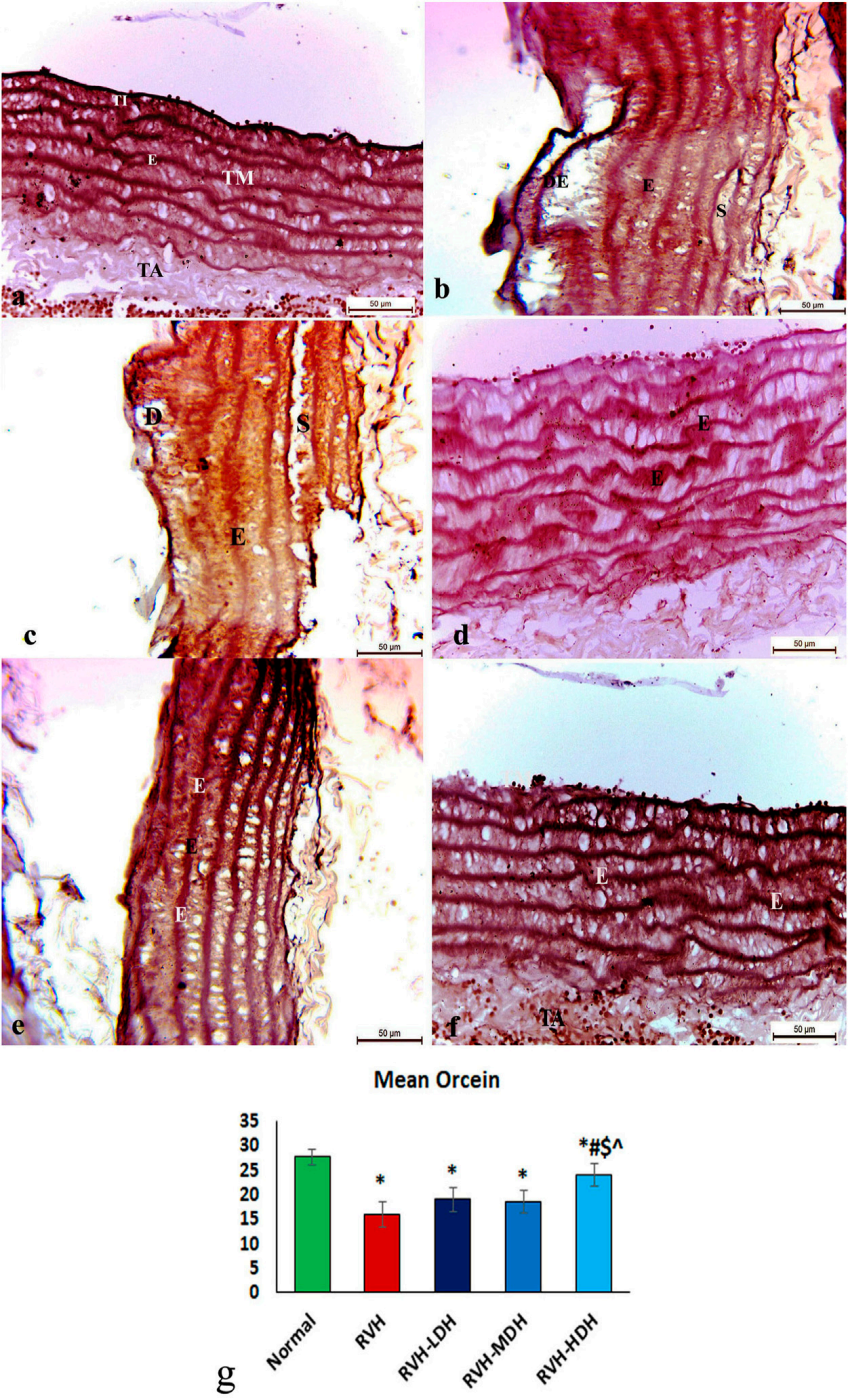
Micrograph of orcein-stained sections of the aortic wall of adult male albino rats. **(A)** Group I: organized and well architectured elastic fibers denoted by “E”. **(B,C)** Group II (RVH): disrupted and loose elastic fibers denoted by “E”. **(D,E)** Groups III (RVH-LDH) and IV (RVH-MDH): slightly preserved elastic fibers. **(F)** Group V (RVH-HDH): well-maintained elastic fibers. **(G)** Demonstrative graph of the mean values of area percentage of elastic fibers (orcein ×400). RVH: renovascular hypertension, LDH: low dose hibiscus, MDH: medium dose of hibiscus, and HDH: high dose of hibiscus. LDH, MDH, and HDH are equivalent to concentrations 5 mg/mL, 10 mg/mL, and 20 mg/mL, respectively. Statistical significance is denoted by * when compared with the control group, # when compared with the RVH group, $ when compared with the LDH, and ^ when compared with the MDH group. Analysis was done using ANOVA, followed by Tukey’s *post hoc* test (n = 7); significance was considered when *p* ˂ 0.05.

The control group presented organized and well-designed elastic fibers (Figure 4A). Although the RVH group indicated disrupted and loose elastic fibers (Figures 4B, C), the RVH-LDH and RVH-MDH groups showed slightly preserved elastic fibers (Figures 4D, E). However, the RVH-HDH group displayed well-maintained elastic fibers (Figure 4F).

**FIGURE 5:**
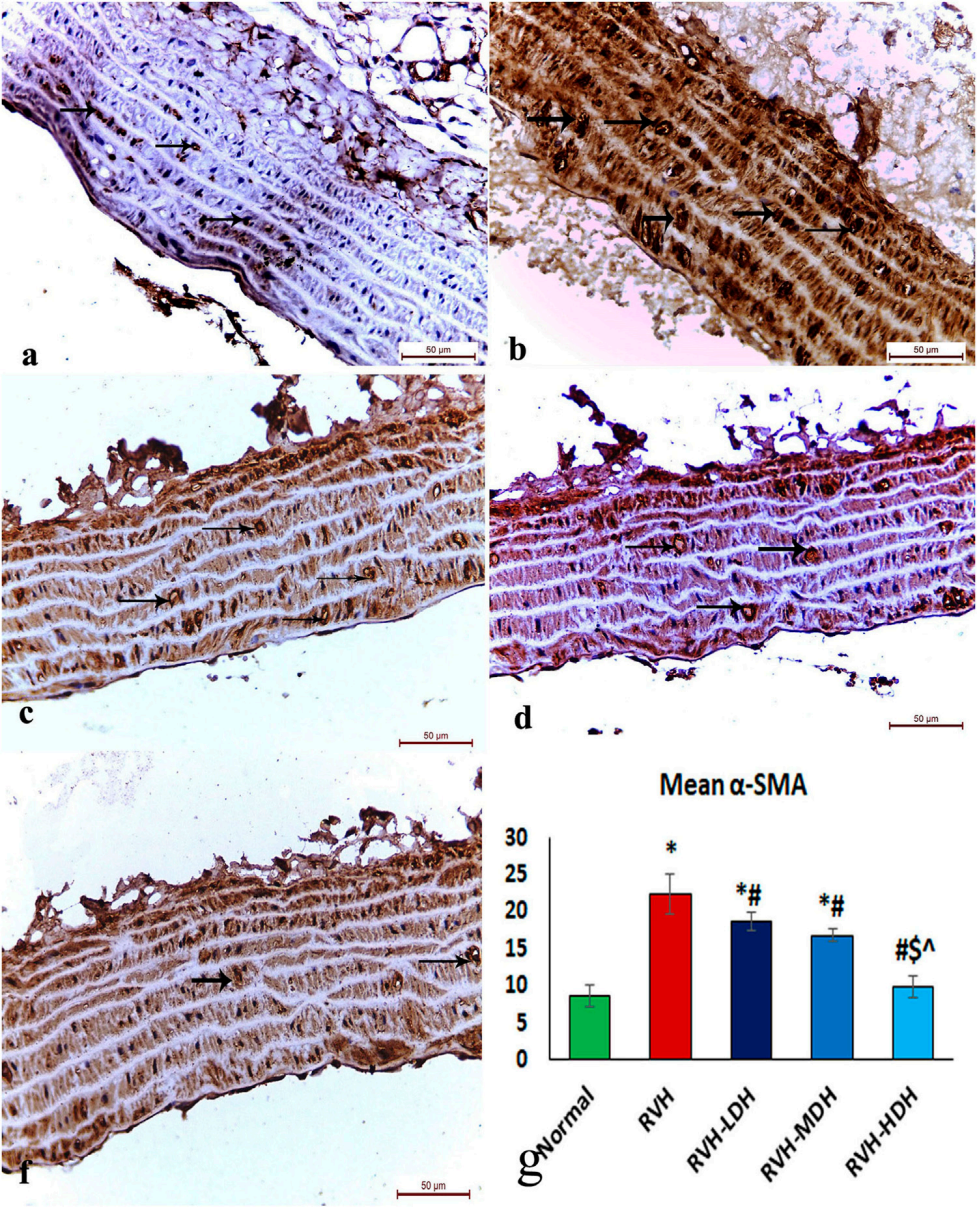
Micrograph of a section of the aortic wall of adult male albino rats exhibiting α-SMA immunohistochemical reaction. **(A)** Group I: trivial reaction, **(B)** group II (RVH): strong, **(C,D)** groups III (RVH-LDH) and IV (RVH-MDH): moderate, **(F)** group V (RVH-HDH): mild. **(G)** Expressive graph of the mean values of area percentage of α-SMA (α-SMA ×400). RVH: renovascular hypertension, LDH: low dose hibiscus, MDH: medium dose of hibiscus, and HDH: high dose of hibiscus. LDH, MDH, and HDH are equivalent to concentrations 5 mg/mL, 10 mg/mL, and 20 mg/mL, respectively. Statistical significance is denoted by * when compared with the control group, # when compared with the RVH group, $ when compared with the LDH, and ^ when compared with the MDH group. Analysis was done using ANOVA, followed by Tukey’s *post hoc* test (n = 7); significance was considered when *p* ˂ 0.05.

**FIGURE 6:**
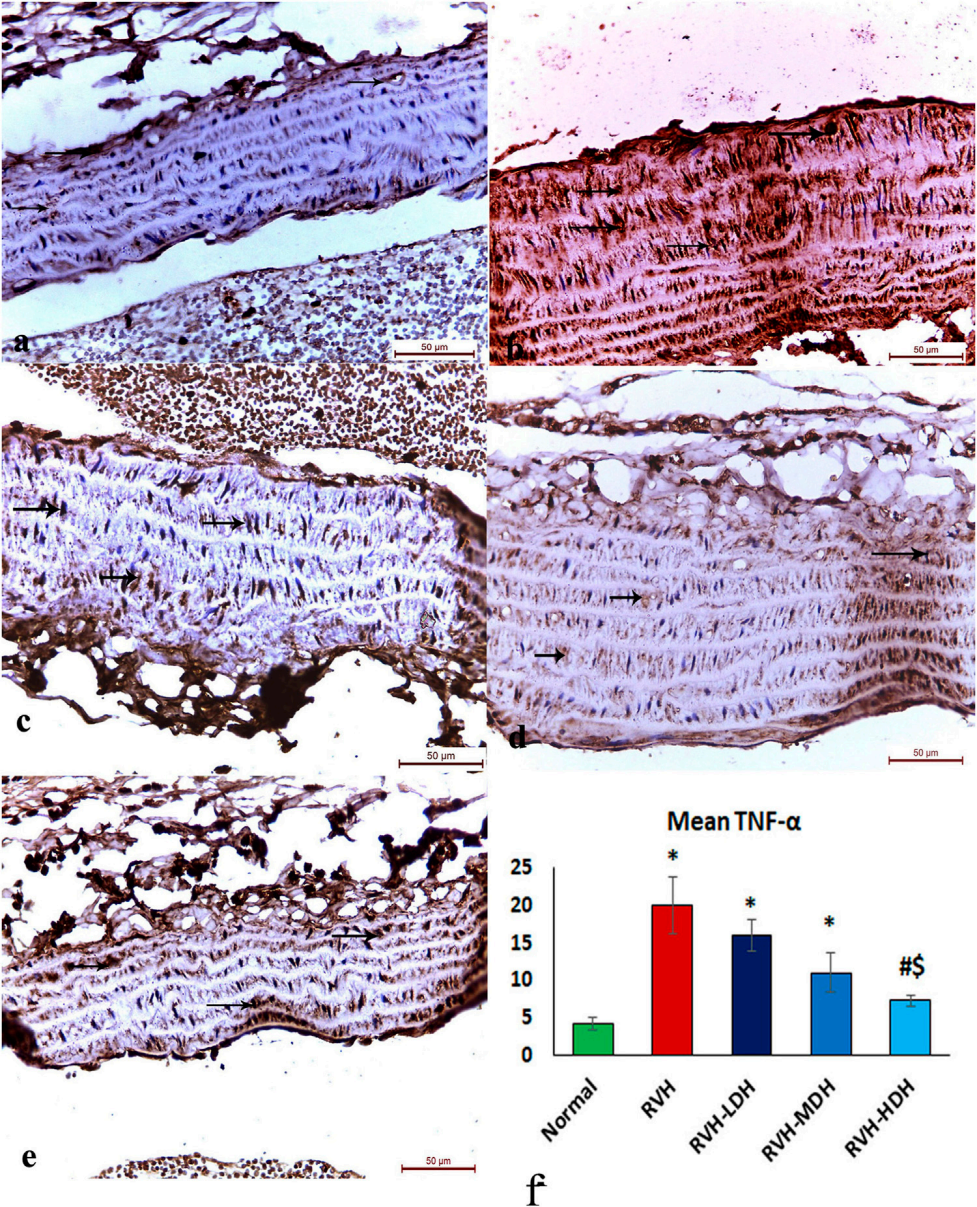
Micrograph of a section of the aortic wall of adult male albino rats exhibiting minimal TNF-α immunohistochemical reaction in groups I **(A)** and II (RVH),**(B)** modest in groups III (RVH-LDH) and IV (RVH-MDH) **(C,D)**, and mild in group V (RVH-HDH) **(E)**. **(F)** Descriptive graph of the mean values of area percentage of TNF-α (TNF-α ×400). RVH: renovascular hypertension, LDH: low dose hibiscus, MDH: medium dose of hibiscus, and HDH: high dose of hibiscus. LDH, MDH, and HDH are equivalent to concentrations 5 mg/mL, 10 mg/mL, and 20 mg/mL, respectively. Statistical significance is denoted by * when compared with the control group, # when compared with the RVH group, $ when compared with the LDH, and ^ when compared with the MDH group. Analysis was done using ANOVA, followed by Tukey’s *post hoc* test (n = 7); significance was considered when *p* ˂ 0.05.

**FIGURE 7:**
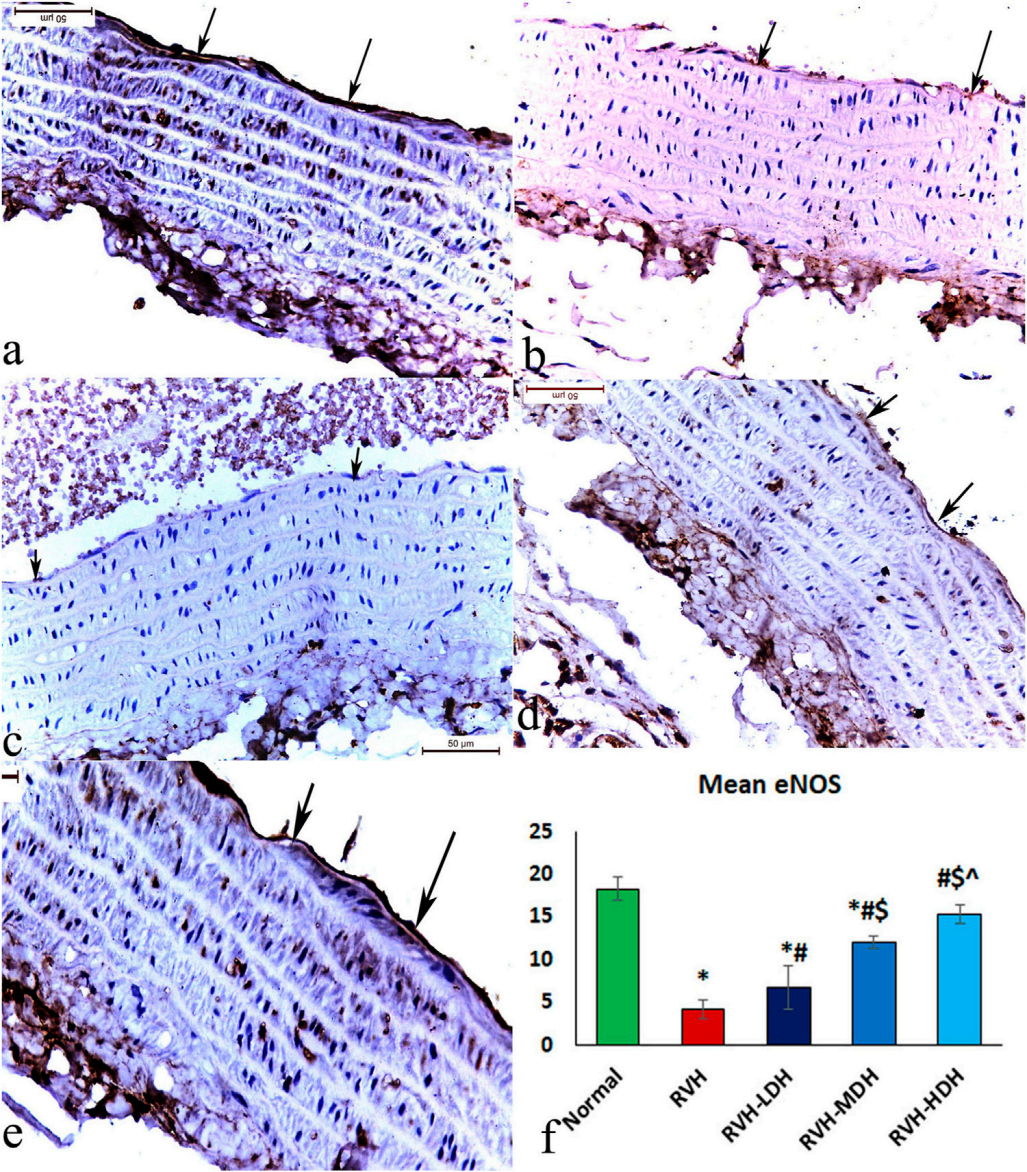
Micrograph of a section of the aortic wall of adult male albino rats exhibiting slight eNOS immunohistochemical reaction in group I **(A)**, strong reaction in group II (RVH) **(B)**, moderate reaction in groups III (RVH-LDH) and IV (RVH-MDH) **(C,D)**, and mild reaction in group V (RVH-HDH) **(E)**. **(F)** Evocative graph of the mean values of area percentage of eNOS (eNOS ×400). RVH: renovascular hypertension, LDH: low dose hibiscus, MDH: medium dose of hibiscus, and HDH: high dose of hibiscus. LDH, MDH, and HDH are equivalent to concentrations 5 mg/mL, 10 mg/mL, and 20 mg/mL, respectively. Statistical significance is denoted by * when compared with the control group, # when compared with the RVH group, $ when compared with the LDH, and ^ when compared with the MDH group. Analysis was done using ANOVA, followed by Tukey’s *post hoc* test (n = 7); significance was considered when *p* ˂ 0.05.

The immunohistochemical reaction of e-NOS showed a down-expression in the RVH group when compared with the control group (Figures 7A, B), whereas it displayed a dose-dependent increase in immune activity in all treated groups (Figures 7C–E). Regarding the α-SMA immune reaction and TNF-α expression, both explicated a trivial reaction in the control group (Figures 56), a strong reaction in the RVH group (Figures 5, 6), a moderate reaction in the RVH-LDH and RVH-MDH groups (Figures 5, 6), and a mild reaction in the RVH-HDH group (Figures 5F, 6).

### 3.5 Morphometric analysis

#### 3.5.1 Mean values of muscle thickness of tunica media, area percent of Masson trichrome, and orcein stains

The mean values of muscle thickness of the tunica media (Figure 2H) of the RVH and RVH-LDH groups revealed statistical significance higher than that of the control group. However, the mean values of muscle thickness in the RVH-LDH, RVH-MDH, and RVH-HDH groups exposed a decrement in statistical significance when compared with that of the RVH group. The mean area percent of collagen fibers in the RVH group revealed a statistically significant increase when compared to that in the control group. However, the area percent in the RVH-LDH, RVH-MDH, and RVH-HDH groups exhibited a statistically significant decrease when compared to that in the RVH group. Conversely, the RVH-HDH group expressed a statistically significant reduction in the mean area percent when compared to that of the RVH-LDH group. Regarding the mean area percent of elastic fibers in the RVH, RVH-LDH, RVH-MDH, and RVH-HDH groups, a statistically significant decline was observed when compared to that in the control group. On the contrary, the RVH-HDH group expressed a statistically significant surge in the area percent of elastic fibers when compared to that in the RVH, RVH-LDH, and RVH-MDH groups (Figure 3G, Figure 4G).

#### 3.5.2 Mean area percent of alpha smooth muscle actin, tumor necrosis factor alpha, and e-NOS

The mean area percent of immune reaction of α-SMA (Figure 5G) in the RVH, RVH-LDH, and RVH-MDH groups displayed a statistically significant increase when compared to that in the control group. On the contrary, the mean area percent of α-SMA in the RVH-LDH, RVH-MDH, and RVH-HDH groups showed a statistically significant decline when compared to that in the RVH group. Meanwhile, the RVH-HDH group showed a statistically significant decrease in the mean area percent of α-SMA when compared to that of the RVH-LDH and RVH-MDH groups.

The mean area percent of immune reaction of TNF-α in the RVH, RVH-LDH, and RVH-MDH groups exhibited a statistically significant increase when compared to that in the control group. On the contrary, the TNF-α mean area percent in the RVH-LDH, RVH-MDH, and RVH-HDH groups expressed a statistically significant decline when compared to that in the RVH group. Furthermore, the RVH-HDH group demonstrated a statistically significant reduction in the TNF-α area percent when compared to that in the RVH-LDH and RVH-MDH groups (Figure 6F).

The mean area percent of immune reaction to e-NOS (Figure 7F) in the RVH, RVH-LDH, and RVH-MDH groups revealed a statistically significant decrease when compared to that in the control group. On the other hand, the mean area percent of e-NOS in the RVH-MDH and RVH-HDH groups displayed a statistically significant upsurge when compared to that in the RVH group. In addition, the RVH-HDH group confirmed a statistically significant increase in the area percent of e-NOS when compared with that in the RVH-LDH and RVH-MDH groups (Figure 7F).

### 3.6 Biochemical results

#### 3.6.1 Hibiscus decreases serum creatinine levels (milligrams per deciliter)

Our model succeeded in inducing hypertension that was associated with a significant increase in serum creatinine level in the RVH group (1.54 ± 0.246) when compared with the sham-operated control group (0.21 ± 0.025). However, the intake of HS in three different concentrations could decrease the serum creatine level in all treated groups when compared with the RVH group. The mean values of creatinine were 0.78 ± 0.2, 0.70 ± 0.025, and 0.51 ± 0.09 for the RVH-LDH, RVH-MDH, and RVH-HDH groups, respectively. Although there was significant decrease in the serum creatinine level in the RVH-HDH group when compared with the RVH-LDH group, the data showed no significant changes in the serum creatinine level in the RVH-HDH group when compared with the RVH-MDH group.

#### 3.6.2 Enhancing production of nitric oxide (NO) and amelioration of increased angiotensin II level and subsequent vascular inflammation by hibiscus dose dependently

Our results showed a significant decrease in the NO level in the RVH group when compared with the control group. However, NO level showed a significant increase in the RVH-LDH, RVH-MDH, and RVH-HDH groups when compared to that in the RVH group. Even more the intake of HDH was able to normalize NO level compared to control group.

Our data showed a significant increase in Ang II levels in the RVH group when compared to that in the control group. Ang II is also considered as a pro-inflammatory factor, and the current data reported a significant increase in VCAM-1 levels in the RVH group when compared with the sham-operated control group. Hibiscus-treated groups showed a significant decrease in Ang II and VCAM-1 levels when compared with the RVH group. The results recorded significant improvements in the abovementioned parameters secondary to the dose-dependent HS treatment (Table 3).

**TABLE 3:** Levels of NO, Ang II, and VCAM-1 in the different experimental groups. LDH, MDH, and HDH are equivalent to concentrations 5 mg/mL, 10 mg/mL, and 20 mg/mL, respectively. Data are presented as mean ± SD. *p* < 0.05 is considered significant. Statistical significance is denoted by * when compared with control, # when compared with RVH, $ when compared with RVH-LDH, ^ when compared with RVH-MDH, and % when compared with RVH-HDH.

Group		NO, nmol/mL	Ang II, pg/mL	VCAM-1, ng/mL
Control	Mean ± SD	27.71 ± 2.4	43.16 ± 3.07	14.87 ± 0.68
RVH	Mean ± SD	8.85 ± 0.54	130.2 ± 3.5	80.59 ± 2.18
		*$^%	*$^%	*$^%
RVH-LDH	Mean ± SD	16.56 ± 2.47	93.04 ± 7.76	53.13 ± 6.98
		*#^%	*#%	*#^%
RVH-MDH	Mean ± SD	21.87 ± 2.88	83.39 ± 7.8	39.37 ± 2.95
		*#$%	*#%	*#$%
RVH-HDH	Mean ± SD	26.16 ± 1.84	56.84 ± 3.23	27.46 ± 4.6
		#$^	*#$^	*#$^

#### 3.6.3 Hibiscus aqueous extract possesses anti-inflammatory capacity and antioxidant effects

**FIGURE 8:**
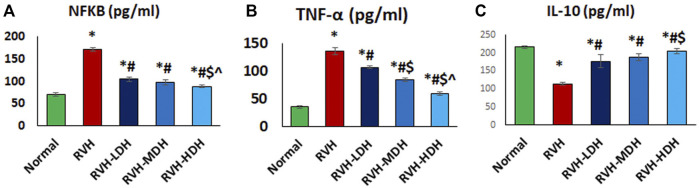
**(A–C)**: Hibiscus aqueous extract employs potent anti-inflammatory effect. The values pro/anti-inflammatory cytokines **(A–C)** in all studied groups were presented as mean ± SD. *p* < 0.05 = significant. *Significant compared to control, # Significant compared RVH, $ Significant compared RVH-LDH, ^ Significant compared RVH-MDH, %Significant compared RVH-HDH. RVH: renovascular hypertension, LDH, low dose hibiscus; MDH, medium-dose hibiscus and HDH high-dose hibiscus. LDH, MDH, and HDH are equivalent to concentrations of 5 mg/ml, 10 mg/ml, and 20 mg/ml respectively. Statistical significance denoted by: *compared to the control group, # compared to the RVH group, $ compared to the LDH, and ^ when compared to the MDH group. Analysis was done using ANOVA followed by Tukey *post hoc* test (n = 7), significance was considered when *p* < 0.05.

A significant reduction in the IL-10 level and elevation in the NF-κB and TNF-α levels were observed in the RVH group when compared with the control group. The obtained results revealed the effectiveness of HS as a potent anti-inflammatory factor by exerting a significant increase in the IL-10 level and decrease in the levels of NF-κB and TNF-α in all treated groups when compared with the RVH group. The effectiveness of protection by HS in all treated groups was evidently dose dependent (Figure 8). Our results showed a significant increase in the MDA and 8-OHdG levels in addition to a significant decrease in the TAC and SOD levels, present as indicators of enhanced lipid peroxidation in the RVH group when compared with the control group. The antioxidant effect of the HS aqueous extract was evident in all the treated groups as a significant decrease in the concentrations of MDA and 8-OHdG, and as an increase in antioxidant factors such as TAC and SOD in the RVH-LDH, RVH-MDH, and RVH-HDH groups compared with the RVH group (Supplementary Table S1).

#### 3.6.4 Hibiscus regulates aortic expression of MALAT1 and cyclophilin A/ERK1/2 axis

**FIGURE 9:**
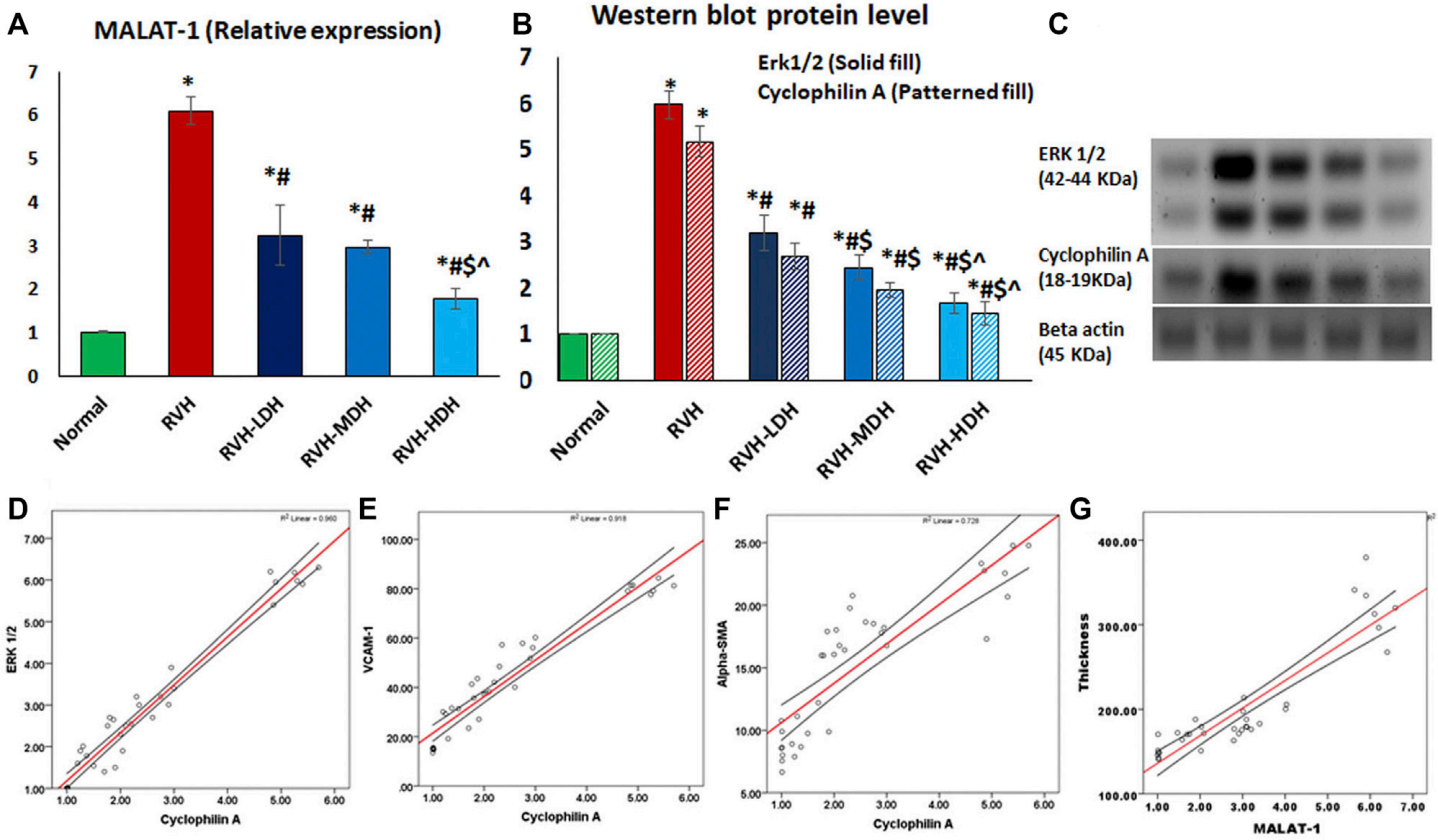
Mean values of expression levels of MALAT and cyclophilin A/ERK1/2 axis **(A,B,C)**. Data are presented as mean ± SD. *p* ˂ 0.05 is considered significant. Statistical significance is denoted by * when compared with the control, # when compared with RVH, $ when compared with RVH-LDH, ^ when compared with RVH-MDH, and % when compared with RVH-HDH. A positive correlation between the mean values of cyclophilin A (CypA) protein levels and the mean values of ERK1/2 protein level (r = 0.980 and *p* ˂ 0.001) **(D)**, VCAM-1 (r = 0.958 and *p* ˂ 0.001) **(E)**, and αSMA (r = 0.853 and *p* ˂ 0.001) **(F)**. A positive correlation between the mean values of the intima media thickness and MALAT1 expression levels (r = 0.906 and *p* ˂ 0.004) **(F)**. RVH: renovascular hypertension, LDH: low dose hibiscus, MDH: medium dose of hibiscus, and HDH: high dose of hibiscus. LDH, MDH, and HDH are equivalent to concentrations 5 mg/mL, 10 mg/mL, and 20 mg/mL, respectively. Statistical significance is denoted by * when compared with the control group, # when compared with the RVH group, $ when compared with the LDH, and ^ when compared with the MDH group. Analysis was done using ANOVA, followed by Tukey’s *post hoc* test (n = 7); significance was considered when *p* ˂ 0.05.

The current results report a significant increase in MALAT1 expression in the RVH group when compared with the control group. The protein level of cyclophilin A (CypA) was significantly increased in the RVH group when compared to that in the control group. The link between CypA and aortic protein levels of ERK1/2 has been previously demonstrated (Satoh et al., 2008). In the current work, the expression level of ERK1/2 was significantly increased in the RVH group when compared with the sham-operated control group. We measured the aortic tissue levels of these parameters in all HS-treated groups, and the data showed a significant dose-dependent decrease in MALAT1 and cyclophilin A/ERK1/2 levels when compared to that in the RVH group (Figures 9A–C).

### 3.7 Correlations

A strong positive correlation existed between the mean values of cyclophilin A (CypA) protein levels on the one side and the mean values of ERK1/2 protein levels (r = 0.980 and *p* ˂ 0.001) (Figure 9D), VCAM-1 (r = 0.958 and *p* ˂ 0.001) (Figure 9E), and α-SMA (r = 0.853 and *p* ˂ 0.001) (Figure 9F) on the other.

A significant positive correlation existed between the thickness of the intima media and the mean values of MALAT1 expression levels (r = 0.906 and *p* ˂ 0.004) (Figure 9G).

## 4 Discussion

The current work studied the role of HS supplementation in attenuating aortopathy that was induced in a rat model of RVH. We performed functional and structural assessments that demonstrated a dose-dependent effective protection of HS.

The modified two-kidney, one-clip (2K1C) Goldblatt model involved renal artery constriction for one kidney and preservation of the other. This model leads to a progressive rise in systolic blood pressure and the development of systemic hypertension (Kim et al., 2016). The 2K1C model has been shown to cause and maintain high arterial blood pressure for several weeks (Lorenz et al., 2011), which is similar to our findings that recorded a successful induction and continuation of hypertension in all the rats exposed to the 2K1C model.

Currently, the vascular response to phenylephrine was enhanced in the RVH group when compared with the control group. Previous studies have related the increased sensitivity of aortic response to vasoconstrictor agents (e.g., norepinephrine) in the 2K1C model to different factors such as a greater release of cytoplasmic Ca^2+^ and activation of protein kinase C (Choi et al., 2014). Other evidences support the hypothesis that ROS serves the pathophysiology of hypertension and impairs endothelium-mediated relaxation (de Miguel et al., 2011; Vanhoutte et al., 2017). ROS, particularly the superoxide anions (O_2_^−^) released in the hypertensive vascular tissues, may cause inactivation of NO and generation of reactive nitrogen species. The availability of NO may have a role in the development of impaired endothelium-mediated relaxation and augmented arterial contractions (Mamun et al., 2020).

Poorly controlled hypertension predisposes to the development of multiple comorbidities, one of which is arterial remodeling (Humphrey, 2021). Interestingly, the primary function of elastic fibers in the arteries is to store the elastic energy during the systole and help recoil during the diastole to allow continuous blood flow. However, sustained elevation of arterial blood pressure induces arterial remodeling. In mice exposed to Ang II infusion for 4 weeks, the thoracic aorta becomes maladapted due to the over-thickening of the vessel wall, mainly secondary to adventitial fibrosis (Spronck et al., 2021).

In this work, the aortic sections of the RVH group showed disruptions in the tunica intima with cellular exfoliation. The endothelial surface is the foremost edge among the walls of the vessels and blood; hence, it is vital for vessel tone and hemostasis (Martinez-Quinones et al., 2018). Regularly, the endothelium adjusts the vasodilator/anticoagulant profusions that hyper-polarize the fundamental vascular smooth muscles to sustain the blood flow.

Secondary to increased blood pressure, a swing in the abovementioned feature’s steadiness, an increment in the release of pro-contractile agents that influence vascular tone (e.g., prostaglandins), and a decrement in the discharge of pro-relaxant influences have been reported (Vaziri et al., 1998).

The pro-hypertensive mediators (i.e., Ang II) interrupt the endothelial barrier (Biancardi et al., 2014). The dysfunctional endothelium exerts a pro-contractile, proinflammatory, and pro-oxidative influence, which is also concomitant with the upsurge of TNF-α in our results. The tunica media revealed a disturbed construction, apparent muscular hypertrophy, and disturbed and torn elastic fibers as also evidenced by orcein stain. The cells appeared degenerated. The reliable verdicts determine that throughout hypertension, hyperplasia and hypertrophy in the VSMCs exemplify two of the vital abnormalities answerable for the inmost vascular altering and consequently amplified progress of the whole peripheral resistance (Szasz and Tostes, 2016).

The significant increase in collagen fibers and α-SMA is an obvious inter-related finding in RVH. The fibrocytes discharge cytokines, metalloproteinases, and the connective tissue matrix’s components (collagen, vimentin, and fibronectin), all of which contribute to tissue restoration (Peng and Herzog, 2012). Nonetheless, in abnormal conditions, for instance increased blood pressure (Wu et al., 2016). Moreover, fibroblasts in the RVH group revealed abrasive sprinkling of collagen fibers, mostly influenced by the occupant fibrocytes, which might also contribute to the vessel wall rigorousness; therefore, fibroblasts play a crucial role in vascular fibrosis caused by an upsurge in type I collagen construction (Wu et al., 2016). Fibroblasts are deliberated as the chief cells for vascular remodeling in response to damage (Yuan et al., 2014).

Stenosis of the renal artery induces ischemia of the kidneys and enhances renin production, which ultimately activates the RAS (Choopani and Nematbakhsh, 2021). In this context, our results showed increased levels of serum Ang II in the RVH group when compared with the control group. Ang II augments lipid peroxidation and ROS production, while the latter correlates to the progression of renal diseases and cardiovascular complications (Mamun et al., 2020). The high levels of Ang II induce VSMC mitogenic actions, enhance DNA and protein synthesis, and may influence vascular hypertrophic and proliferative actions (Moinuddin et al., 2013). Ang II scavenges NO and disrupts vascular relaxation (Mamun et al., 2020). This supports our results which reveal significantly increased Ang II, and decreased NO level and e-NOS immunostaining in the RVH group when compared with the control group.

Creatinine levels were used as the kidney damage marker, and the results showed that the levels of creatinine were significantly increased in the RVH group when compared to that in the control group, which is in accordance to Mamun et al. (2020) who reported that the possible mechanisms underlying decreased kidney function are the excess production of ROS and over-activation of the RAS.

TNF-α could activate endothelial cells, and similar to the effects of Ang II, it enhances cell survival, migration, and differentiation in the aorta of 2K1C (Zhang et al., 2017). NF-κB is the key regulator of TNF-α gene activation. After being activated, NF-κB undergoes nuclear translocation and induces the transcription of various pro-inflammatory genes, such as TNF-α. Whereas the anti-inflammatory factor IL-10 inhibits the production of pro-inflammatory cytokines in the human monocytes through the suppression of NF-κB activation (Dhingra et al., 2009), Ang II activates NF-κB that mediates the production of cell adhesion molecules, such as VCAM-1 (Pothen et al., 2022). In this context, the RVH group showed a significant increase in TNF-α, NFк-B, and VCAM-1 together with a significant decrease in IL-10 levels.

Both MDA and 8-OHdG levels are important biomarkers of oxidative stress (Mamun et al., 2020). Unlike free radical-scavenging antioxidant factors (SOD and TAC) that were markedly reduced, the pro-oxidant factor levels (MDA and 8-OHdG) were significantly increased in the RVH group when compared with the control group.

Several antihypertensive mechanisms are linked to the actions of HS. HS could exert diuretic and vasodilating activities, and inhibition of angiotensin converting enzyme (Salem et al., 2022). Our data reported the influence of HS on controlling arterial blood pressure. Moreover, the Ang II levels were significantly decreased in all HS-treated groups dose dependently when compared to that in the RVH group.

The HS-treated groups showed a significant increase in the NO level when compared with the RVH group, and this increase was associated with improved immunostaining of e-NOS in all the treated groups in a dose-dependent manner when compared with the RVH group. Our results support that which was previously reported by Lim et al. (2017) who described the HS hypotensive mechanism through its ability to augment endothelial NO-cGMP (cyclic guanosine monophosphate) pathway.

RVH-HS-treated groups revealed amelioration of histopathological alterations. The administration of HS was established to recover oxidative stress, which is related to declining ROS production. Rendering to prior studies, the anti-oxidant proxy would shield the aortic structure from the promoted oxidative damage (Rietjens et al., 2007). It can hinder the ultra-structural vagaries of the aorta, consequently avoiding the expansion of atherosclerotic alterations. Moreover, HS adjusts Ca^2+^ entrance pathways and contractile proteins in the contraction signaling pathway (Lim et al., 2017). Administration of HS increased the expressions of e-NOS in the aorta.

Hibiscus intake for a month modified organ renovation by enhancing anti-oxidant capacity and restraining hypertrophy and fibrosis (Si et al., 2019). The current work reports a significant increase in MALAT1 expression in the RVH group and its decrease in the HS-treated groups. This is concomitant with the findings of Xue et al. (2019), who postulated that MALAT1 was extremely elevated in hypertensive cases and that the release of inflammatory mediators, comprising TNF-α, IL-1β, and IL-6, could be repressed by shutting down MALAT1. Consequently, there was a significant positive correlation between smooth muscle thickness and MALAT1 in our study. Consistent with previous observation (Wang et al., 2019), MALAT1 was over-expressed in the thoracic aortic vascular tissues of hypertensive rats, whereas MALAT1 shutdown resulted in accelerated migration of endothelial cells (Michalik et al., 2014). MALAT1 became convoluted in interchanging the phenotype of VSMCs. Downregulation of MALAT1 endorsed the conversion of smooth muscle cells from proliferative to differentiated phenotypes. Therefore, the results of shutting down MALAT1 in the VSMCs are related to the significant arrest of the cell cycle (Song et al., 2018). Moreover, Wang et al. (2019) postulated that MALAT1 levels were significantly upregulated in the pulmonary arteries of patients with pulmonary hypertension that can be matched to the pulmonary arteries in the control group.

In the current study, we report increased CyPA protein levels (as an oxidative stress–induced factor) in the RVH group when compared to that of the control group. Moreover, our data shows a strong positive correlation between CyPA on the one side and the VCAM-1 and ERK1/2 protein levels on the other side.

CyPA mediates ERK1/2 phosphorylation, enhances VSMC proliferation (Satoh et al., 2009), and increases the expression of VCAM-1 and E-selectin. Moreover, the secreted CyPA stimulates different pathways, such as ERK1/2, which, in turn, contribute to ROS production (Satoh, 2014). In a synergistic way, Ang II induces Rho-kinase-mediated CyPA secretion, which eventually augments oxidative stress (Satoh et al., 2009).

The widely used HS in beverage industries and as food colorant (Hirunpanich et al., 2006) contains different chemical constituents such as polyphenolic compounds (i.e., gallic acid, rutin, and quercetin), particularly in the calyces (Singh et al., 2021; Banwo et al., 2022). These estimated compounds act as potent anti-oxidant factors and significantly reduce oxidative stress in rat hepatocytes (Hirunpanich et al., 2006).

The current study highlights the importance of the old herbal medicine, HS, as an antioxidant and anti-inflammatory mediator in attenuating aortopathy. Applying different HS doses was our approach, and investigating its molecular target, particularly CyPA, which to the best of our knowledge is being investigated in 2K1C models for the first time, was our aim. HS succeeded in downregulating CyPA/ERK1/2 protein levels. Additional work is highly recommended. Thus, we are conducting a complementary work on the most active ingredient that may influence aortic remodeling changes in RVH.

## 5 Conclusion

**FIGURE 10:**
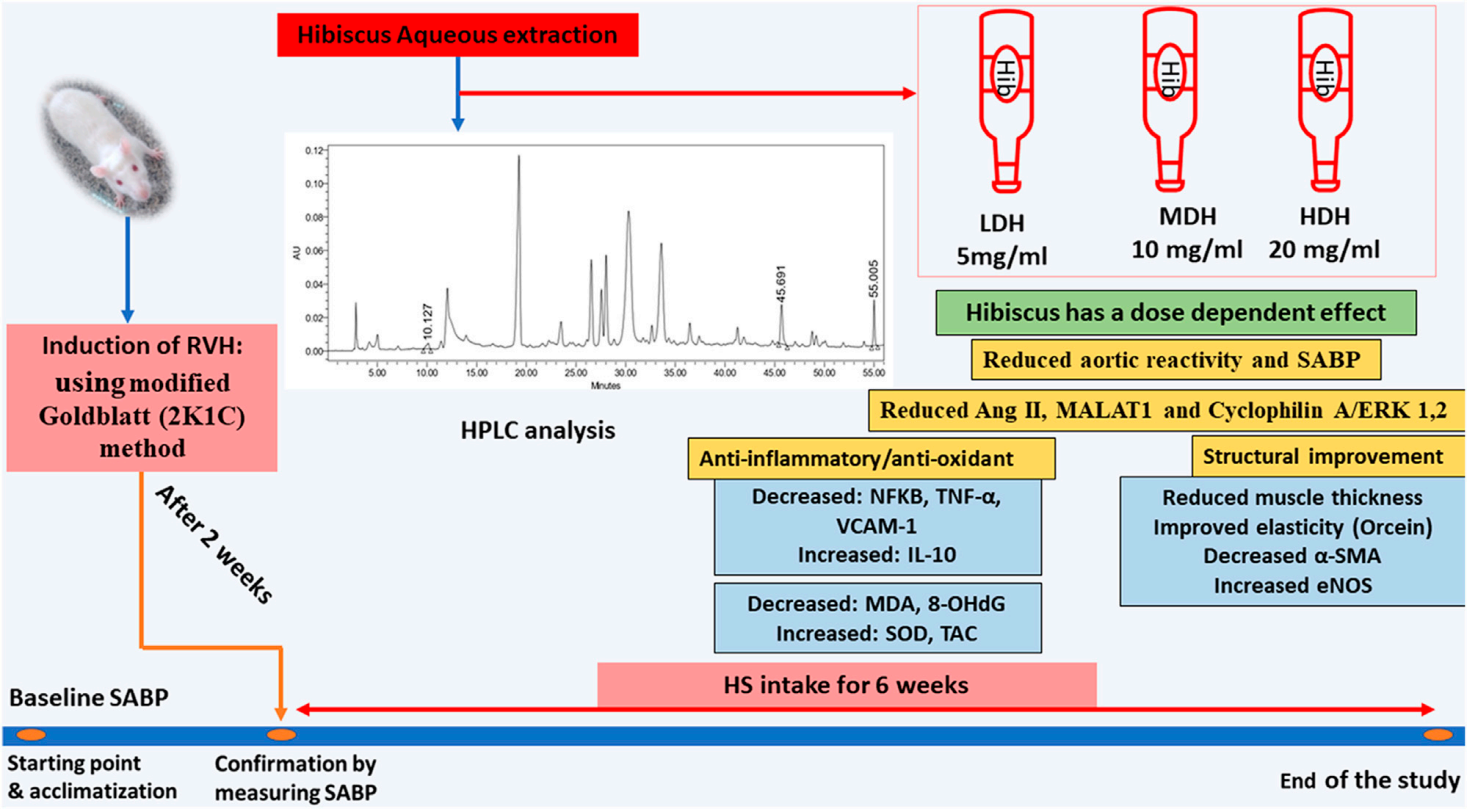
Schematic presentation demonstrating the beneficial effects of *Hibiscus sabdariffa* L. supplementation in ameliorating renovascular hypertension-induced aortopathy. Following hibiscus preparation and high-performance liquid chromatography (HPLC) analysis, three concentrations were prepared to produce low, medium, and high doses of hibiscus to get concentrations of 5 mg/mL, 10 mg/mL, and 20 mg/mL, respectively. Hibiscus supplementation modified aortic renovation by decreasing systolic arterial blood pressure (SABP) sensitivity of aortic response to phenylephrine, enhancing anti-inflammatory and antioxidant capacities, and restraining aortic hypertrophy and fibrosis. Hibiscus intake was able to decrease angiotensin II levels (Ang II) and downregulate metastasis-associated lung adenocarcinoma transcript (MALAT1) and cyclophilin A (CyPA)/ERK1/2 protein levels dose dependently.

Adding to its multiple beneficial effects, the HS aqueous extract succeeded in ameliorating vascular pro-inflammatory and pro-oxidant functions, while decreasing aortic MALAT1 and CyPA/ERK1/2 protein levels (demonstrated in Figure 10). Furthermore, the results of the current work provide a new mechanistic basis for HS action and participate in the understanding of the mechanisms behind the inhibition of VSMC proliferation induced by the 2K1C model.

## Data Availability

The original contributions presented in the study are included in the article/Supplementary Material, further inquiries can be directed to the corresponding authors..
