# Supplementary material for: Hibiscus attenuates renovascular hypertension–induced aortic remodeling dose dependently: the oxidative stress role and Ang II/cyclophilin A/ERK1/2 signaling
Source: Front Physiol. 2023 Jun 21;14:1116705. doi: 10.3389/fphys.2023.1116705 (PMC10321301; doi:10.3389/fphys.2023.1116705)
Supplement: Supplementary file 1 [file Table1.DOCX]

**Supplementary table 1:** The levels of oxidative stress markers in the different experimental groups. Data presented as mean ±SD. P< 0.05 = significant. *Significant compared to control, # Significant compared RVH, $ Significant compared RVH-LDH, ^ Significant compared RVH-MDH, % Significant compared RVH-HDH.

| Groups | | MDA  nmol/gm tissue | 8-OHdG  nmol/gm tissue | TAC  nmol/gm tissue | SOD  U/min/gm tissue |
| --- | --- | --- | --- | --- | --- |
| Control | Mean ±SD | 33.80±1.25 | 5.27±0.3 | 117.70±4.08 | 91±6.89 |
| RVH | Mean ±SD | 152.79±5.03  *$^% | 16.79±1.22  *$^% | 57.06±4.6  *$^% | 30.88±1.65  *$^% |
| RVH- LDH | Mean ±SD | 83.96±12.36  *#^% | 9.64±0.73  *#^% | 82±1.79  *#% | 57.69±11.2  *#% |
| RVH- MDH | Mean ±SD | 67.50±9.91  *#$% | 7.40±0.32  *#$ | 88.37±5.75  *#$% | 66.46±5.9  *#% |
| RVH- HDH | Mean ±SD | 49.10±4.18  *#$^ | 6.13±0.16  #$ | 109±3.56  *#$^ | 81.59±4.5  #$^ |
