# Supplementary material for: Hibiscus attenuates renovascular hypertension–induced aortic remodeling dose dependently: the oxidative stress role and Ang II/cyclophilin A/ERK1/2 signaling
Source: Front Physiol. 2023 Jun 21;14:1116705. doi: 10.3389/fphys.2023.1116705 (PMC10321301; doi:10.3389/fphys.2023.1116705)
Supplement: Supplementary file 2 [file Table2.DOCX]

**Hibiscus Attenuates** **Renovascular Hypertension-induced Aortic Remodeling Dose Dependently: The Role of Oxidative Stress and Ang II/** **Cyclophilin A/ERK Signaling**

**Asmaa Mohammed ShamsEldeen^1^*,** **Ahmed Fawzy^1^,** **Hend Ashour ^3,1^ , Marwa Abdel-Rahman ^1^, Hend Elsayed Nasr^5^, Lina Abdelhady Mohammed^5^, Noha Samir Abdel Latif ^4^, Shereen Abdelfattah^2^**

^1^Department of Physiology, Faculty of Medicine, Cairo University, Egypt

^2^Department of Anatomy and Embryology Faculty of Medicine, Cairo University, Egypt

^3^Department of Physiology, Faculty of Medicine, King Khalid University, Abha, Saudi Arabia

^4^Department of Pharmacology, Faculty of Medicine, Cairo University, Egypt

^5^Department of Medical Biochemistry and Molecular Biology, Benha University, Egypt

| **Tests of Normality** | | | | | | | | | | | | |  |
| --- | --- | --- | --- | --- | --- | --- | --- | --- | --- | --- | --- | --- | --- |
|  | | Group | | Kolmogorov-Smirnov^a^ | | | | | | Shapiro-Wilk | | |  |
|  | |  |  | Statistic | | df | Sig. | | | Statistic | df | Sig. |  |
| TG | | Control | | .271 | | 7 | .129 | | | .828 | 7 | .077 |  |
|  |  | RVH | | .291 | | 7 | .076 | | | .896 | 7 | .309 |  |
|  |  | RVH-LDH | | .230 | | 7 | .200^*^ | | | .953 | 7 | .753 |  |
|  |  | RVH-MDH | | .243 | | 7 | .200^*^ | | | .917 | 7 | .449 |  |
|  |  | RVH-HDH | | .225 | | 7 | .200^*^ | | | .848 | 7 | .117 |  |
| TC | | Control | | .151 | | 7 | .200^*^ | | | .930 | 7 | .550 |  |
|  |  | RVH | | .293 | | 7 | .070 | | | .844 | 7 | .109 |  |
|  |  | RVH-LDH | | .262 | | 7 | .160 | | | .864 | 7 | .165 |  |
|  |  | RVH-MDH | | .289 | | 7 | .079 | | | .817 | 7 | .060 |  |
|  |  | RVH-HDH | | .192 | | 7 | .200^*^ | | | .890 | 7 | .274 |  |
| HDL | | Control | | .153 | | 7 | .200^*^ | | | .970 | 7 | .897 |  |
|  |  | RVH | | .312 | | 7 | .038 | | | .876 | 7 | .209 |  |
|  |  | RVH-LDH | | .226 | | 7 | .200^*^ | | | .888 | 7 | .263 |  |
|  |  | RVH-MDH | | .230 | | 7 | .200^*^ | | | .841 | 7 | .101 |  |
|  |  | RVH-HDH | | .244 | | 7 | .200^*^ | | | .905 | 7 | .359 |  |
| Creatinine | | Control | | .229 | | 7 | .200^*^ | | | .871 | 7 | .190 |  |
|  |  | RVH | | .209 | | 7 | .200^*^ | | | .958 | 7 | .803 |  |
|  |  | RVH-LDH | | .235 | | 7 | .200^*^ | | | .860 | 7 | .151 |  |
|  |  | RVH-MDH | | .226 | | 7 | .200^*^ | | | .865 | 7 | .166 |  |
|  |  | RVH-HDH | | .249 | | 7 | .200^*^ | | | .934 | 7 | .581 |  |
| NO | | Control | | .277 | | 7 | .113 | | | .826 | 7 | .073 |  |
|  |  | RVH | | .259 | | 7 | .173 | | | .901 | 7 | .339 |  |
|  |  | RVH-LDH | | .266 | | 7 | .145 | | | .927 | 7 | .525 |  |
|  |  | RVH-MDH | | .267 | | 7 | .142 | | | .892 | 7 | .285 |  |
|  |  | RVH-HDH | | .224 | | 7 | .200^*^ | | | .872 | 7 | .194 |  |
| MDA | | Control | | .216 | | 7 | .200^*^ | | | .882 | 7 | .238 |  |
|  |  | RVH | | .292 | | 7 | .073 | | | .816 | 7 | .058 |  |
|  |  | RVH-LDH | | .218 | | 7 | .200^*^ | | | .966 | 7 | .867 |  |
|  |  | RVH-MDH | | .238 | | 7 | .200^*^ | | | .860 | 7 | .150 |  |
|  |  | RVH-HDH | | .203 | | 7 | .200^*^ | | | .932 | 7 | .566 |  |
| 8DG | | Control | | .180 | | 7 | .200^*^ | | | .924 | 7 | .505 |  |
|  |  | RVH | | .256 | | 7 | .184 | | | .893 | 7 | .290 |  |
|  |  | RVH-LDH | | .209 | | 7 | .200^*^ | | | .907 | 7 | .373 |  |
|  |  | RVH-MDH | | .196 | | 7 | .200^*^ | | | .949 | 7 | .722 |  |
|  |  | RVH-HDH | | .297 | | 7 | .062 | | | .858 | 7 | .144 |  |
| TAC | | Control | | .195 | | 7 | .200^*^ | | | .960 | 7 | .821 |  |
|  |  | RVH | | .287 | | 7 | .084 | | | .905 | 7 | .361 |  |
|  |  | RVH-LDH | | .244 | | 7 | .200^*^ | | | .875 | 7 | .206 |  |
|  |  | RVH-MDH | | .292 | | 7 | .072 | | | .903 | 7 | .348 |  |
|  |  | RVH-HDH | | .168 | | 7 | .200^*^ | | | .934 | 7 | .584 |  |
| SOD | | Control | | .119 | | 7 | .200^*^ | | | .991 | 7 | .995 |  |
|  |  | RVH | | .216 | | 7 | .200^*^ | | | .925 | 7 | .510 |  |
|  |  | RVH-LDH | | .268 | | 7 | .137 | | | .870 | 7 | .185 |  |
|  |  | RVH-MDH | | .287 | | 7 | .085 | | | .843 | 7 | .107 |  |
|  |  | RVH-HDH | | .260 | | 7 | .168 | | | .884 | 7 | .246 |  |
| Renin | | Control | | .172 | | 7 | .200^*^ | | | .953 | 7 | .754 |  |
|  |  | RVH | | .269 | | 7 | .135 | | | .799 | 7 | .040 |  |
|  |  | RVH-LDH | | .202 | | 7 | .200^*^ | | | .882 | 7 | .237 |  |
|  |  | RVH-MDH | | .191 | | 7 | .200^*^ | | | .924 | 7 | .498 |  |
|  |  | RVH-HDH | | .219 | | 7 | .200^*^ | | | .836 | 7 | .091 |  |
| Angiotensin | | Control | | .167 | | 7 | .200^*^ | | | .970 | 7 | .897 |  |
|  |  | RVH | | .196 | | 7 | .200^*^ | | | .895 | 7 | .300 |  |
|  |  | RVH-LDH | | .192 | | 7 | .200^*^ | | | .897 | 7 | .316 |  |
|  |  | RVH-MDH | | .217 | | 7 | .200^*^ | | | .873 | 7 | .197 |  |
|  |  | RVH-HDH | | .205 | | 7 | .200^*^ | | | .943 | 7 | .666 |  |
| VCAM-1 | | Control | | .255 | | 7 | .186 | | | .859 | 7 | .147 |  |
|  |  | RVH | | .211 | | 7 | .200^*^ | | | .941 | 7 | .646 |  |
|  |  | RVH-LDH | | .236 | | 7 | .200^*^ | | | .893 | 7 | .292 |  |
|  |  | RVH-MDH | | .226 | | 7 | .200^*^ | | | .925 | 7 | .511 |  |
|  |  | RVH-HDH | | .227 | | 7 | .200^*^ | | | .875 | 7 | .205 |  |
| NFKB | | Control | | .178 | | 7 | .200^*^ | | | .904 | 7 | .357 |  |
|  |  | RVH | | .206 | | 7 | .200^*^ | | | .920 | 7 | .468 |  |
|  |  | RVH-LDH | | .168 | | 7 | .200^*^ | | | .940 | 7 | .643 |  |
|  |  | RVH-MDH | | .226 | | 7 | .200^*^ | | | .939 | 7 | .631 |  |
|  |  | RVH-HDH | | .249 | | 7 | .200^*^ | | | .869 | 7 | .184 |  |
| TNF | | Control | | .161 | | 7 | .200^*^ | | | .961 | 7 | .831 |  |
|  |  | RVH | | .175 | | 7 | .200^*^ | | | .976 | 7 | .938 |  |
|  |  | RVH-LDH | | .274 | | 7 | .122 | | | .849 | 7 | .120 |  |
|  |  | RVH-MDH | | .217 | | 7 | .200^*^ | | | .915 | 7 | .434 |  |
|  |  | RVH-HDH | | .219 | | 7 | .200^*^ | | | .935 | 7 | .598 |  |
| IL10 | | Control | | .200 | | 7 | .200^*^ | | | .972 | 7 | .911 |  |
|  |  | RVH | | .102 | | 7 | .200^*^ | | | .996 | 7 | 1.000 |  |
|  |  | RVH-LDH | | .174 | | 7 | .200^*^ | | | .957 | 7 | .791 |  |
|  |  | RVH-MDH | | .166 | | 7 | .200^*^ | | | .947 | 7 | .706 |  |
|  |  | RVH-HDH | | .159 | | 7 | .200^*^ | | | .963 | 7 | .843 |  |
| MALAT-1 | | Control | | .172 | | 7 | .200^*^ | | | .967 | 7 | .873 |  |
|  |  | RVH | | .161 | | 7 | .200^*^ | | | .979 | 7 | .953 |  |
|  |  | RVH-LDH | | .235 | | 7 | .200^*^ | | | .902 | 7 | .341 |  |
|  |  | RVH-MDH | | .156 | | 7 | .200^*^ | | | .955 | 7 | .775 |  |
|  |  | RVH-HDH | | .136 | | 7 | .200^*^ | | | .966 | 7 | .867 |  |
| PE10 | | Control | | .267 | | 7 | .140 | | | .894 | 7 | .294 |  |
|  |  | RVH | | .287 | | 7 | .084 | | | .855 | 7 | .135 |  |
|  |  | RVH-LDH | | .119 | | 7 | .200^*^ | | | .967 | 7 | .873 |  |
|  |  | RVH-MDH | | .256 | | 7 | .182 | | | .833 | 7 | .086 |  |
|  |  | RVH-HDH | | .191 | | 7 | .200^*^ | | | .947 | 7 | .699 |  |
| PE20 | | Control | | .173 | | 7 | .200^*^ | | | .922 | 7 | .482 |  |
|  |  | RVH | | .222 | | 7 | .200^*^ | | | .905 | 7 | .365 |  |
|  |  | RVH-LDH | | .175 | | 7 | .200^*^ | | | .906 | 7 | .372 |  |
|  |  | RVH-MDH | | .207 | | 7 | .200^*^ | | | .959 | 7 | .813 |  |
|  |  | RVH-HDH | | .159 | | 7 | .200^*^ | | | .974 | 7 | .923 |  |
| PE40 | | Control | | .181 | | 7 | .200^*^ | | | .880 | 7 | .226 |  |
|  |  | RVH | | .181 | | 7 | .200^*^ | | | .924 | 7 | .505 |  |
|  |  | RVH-LDH | | .182 | | 7 | .200^*^ | | | .961 | 7 | .827 |  |
|  |  | RVH-MDH | | .180 | | 7 | .200^*^ | | | .863 | 7 | .162 |  |
|  |  | RVH-HDH | | .221 | | 7 | .200^*^ | | | .909 | 7 | .389 |  |
| ABP | | Control | | .214 | | 7 | .200^*^ | | | .938 | 7 | .617 |  |
|  |  | RVH | | .214 | | 7 | .200^*^ | | | .848 | 7 | .117 |  |
|  |  | RVH-LDH | | .214 | | 7 | .200^*^ | | | .933 | 7 | .573 |  |
|  |  | RVH-MDH | | .182 | | 7 | .200^*^ | | | .920 | 7 | .471 |  |
|  |  | RVH-HDH | | .237 | | 7 | .200^*^ | | | .896 | 7 | .307 |  |
| ABP-2W | | Control | | .286 | | 7 | .087 | | | .869 | 7 | .181 |  |
|  |  | RVH | | .216 | | 7 | .200^*^ | | | .891 | 7 | .278 |  |
|  |  | RVH-LDH | | .184 | | 7 | .200^*^ | | | .934 | 7 | .586 |  |
|  |  | RVH-MDH | | .177 | | 7 | .200^*^ | | | .939 | 7 | .633 |  |
|  |  | RVH-HDH | | .236 | | 7 | .200^*^ | | | .937 | 7 | .615 |  |
| ABP- Final | | Control | | .190 | | 7 | .200^*^ | | | .964 | 7 | .849 |  |
|  |  | RVH | | .278 | | 7 | .110 | | | .887 | 7 | .258 |  |
|  |  | RVH-LDH | | .201 | | 7 | .200^*^ | | | .917 | 7 | .443 |  |
|  |  | RVH-MDH | | .249 | | 7 | .200^*^ | | | .910 | 7 | .395 |  |
|  |  | RVH-HDH | | .211 | | 7 | .200^*^ | | | .904 | 7 | .357 |  |
| MT | | Control | | .177 | | 7 | .200^*^ | | | .960 | 7 | .820 |  |
|  |  | RVH | | .128 | | 7 | .200^*^ | | | .993 | 7 | .997 |  |
|  |  | RVH-LDH | | .253 | | 7 | .194 | | | .909 | 7 | .388 |  |
|  |  | RVH-MDH | | .292 | | 7 | .072 | | | .859 | 7 | .147 |  |
|  |  | RVH-HDH | | .170 | | 7 | .200^*^ | | | .968 | 7 | .881 |  |
| Thickness | | Control | | .298 | | 7 | .060 | | | .796 | 7 | .037 |  |
|  |  | RVH | | .148 | | 7 | .200^*^ | | | .990 | 7 | .993 |  |
|  |  | RVH-LDH | | .206 | | 7 | .200^*^ | | | .891 | 7 | .281 |  |
|  |  | RVH-MDH | | .264 | | 7 | .149 | | | .890 | 7 | .273 |  |
|  |  | RVH-HDH | | .267 | | 7 | .141 | | | .909 | 7 | .389 |  |
| Orcein | | Control | | .190 | | 7 | .200^*^ | | | .967 | 7 | .876 |  |
|  |  | RVH | | .121 | | 7 | .200^*^ | | | .991 | 7 | .995 |  |
|  |  | RVH-LDH | | .125 | | 7 | .200^*^ | | | .992 | 7 | .997 |  |
|  |  | RVH-MDH | | .231 | | 7 | .200^*^ | | | .922 | 7 | .488 |  |
|  |  | RVH-HDH | | .219 | | 7 | .200^*^ | | | .926 | 7 | .516 |  |
| TNF-Histology | | Control | | .226 | | 7 | .200^*^ | | | .953 | 7 | .753 |  |
|  |  | RVH | | .165 | | 7 | .200^*^ | | | .976 | 7 | .936 |  |
|  |  | RVH-LDH | | .160 | | 7 | .200^*^ | | | .949 | 7 | .720 |  |
|  |  | RVH-MDH | | .221 | | 7 | .200^*^ | | | .934 | 7 | .587 |  |
|  |  | RVH-HDH | | .217 | | 7 | .200^*^ | | | .948 | 7 | .713 |  |
| Alpha-SMA | | Control | | .200 | | 7 | .200^*^ | | | .973 | 7 | .918 |  |
|  |  | RVH | | .252 | | 7 | .200^*^ | | | .876 | 7 | .210 |  |
|  |  | RVH-LDH | | .210 | | 7 | .200^*^ | | | .973 | 7 | .918 |  |
|  |  | RVH-MDH | | .212 | | 7 | .200^*^ | | | .813 | 7 | .055 |  |
|  |  | RVH-HDH | | .185 | | 7 | .200^*^ | | | .961 | 7 | .831 |  |
| eNOS | | Control | | .196 | | 7 | .200^*^ | | | .955 | 7 | .778 |  |
|  |  | RVH | | .135 | | 7 | .200^*^ | | | .986 | 7 | .982 |  |
|  |  | RVH-LDH | | .271 | | 7 | .129 | | | .880 | 7 | .227 |  |
|  |  | RVH-MDH | | .235 | | 7 | .200^*^ | | | .837 | 7 | .092 |  |
|  |  | RVH-HDH | | .150 | | 7 | .200^*^ | | | .948 | 7 | .715 |  |
| BW (initial) | Control | | .151 | | 7 | | | .200^*^ | .983 | | 7 | .971 | |
|  | RVH | | .152 | | 7 | | | .200^*^ | .948 | | 7 | .713 | |
|  | RVH-LDH | | .198 | | 7 | | | .200^*^ | .946 | | 7 | .695 | |
|  | RVH-MDH | | .128 | | 7 | | | .200^*^ | .990 | | 7 | .992 | |
|  | RVH-HDH | | .268 | | 7 | | | .139 | .773 | | 7 | .022 | |
| BW (2w) | Control | | .229 | | 7 | | | .200^*^ | .917 | | 7 | .443 | |
|  | RVH | | .164 | | 7 | | | .200^*^ | .959 | | 7 | .810 | |
|  | RVH-LDH | | .272 | | 7 | | | .128 | .852 | | 7 | .127 | |
|  | RVH-MDH | | .243 | | 7 | | | .200^*^ | .904 | | 7 | .357 | |
|  | RVH-HDH | | .270 | | 7 | | | .132 | .852 | | 7 | .129 | |
| BW (4w) | Control | | .189 | | 7 | | | .200^*^ | .936 | | 7 | .599 | |
|  | RVH | | .199 | | 7 | | | .200^*^ | .961 | | 7 | .824 | |
|  | RVH-LDH | | .247 | | 7 | | | .200^*^ | .887 | | 7 | .257 | |
|  | RVH-MDH | | .325 | | 7 | | | .025 | .847 | | 7 | .114 | |
|  | RVH-HDH | | .177 | | 7 | | | .200^*^ | .934 | | 7 | .588 | |
| BW (6w) | Control | | .209 | | 7 | | | .200^*^ | .912 | | 7 | .407 | |
|  | RVH | | .114 | | 7 | | | .200^*^ | .987 | | 7 | .985 | |
|  | RVH-LDH | | .293 | | 7 | | | .070 | .841 | | 7 | .101 | |
|  | RVH-MDH | | .300 | | 7 | | | .056 | .834 | | 7 | .086 | |
|  | RVH-HDH | | .273 | | 7 | | | .200^*^ | .837 | | 7 | .094 | |
| BW (8w) | Control | | .151 | | 7 | | | .200^*^ | .983 | | 7 | .971 | |
|  | RVH | | .148 | | 7 | | | .200^*^ | .949 | | 7 | .720 | |
|  | RVH-LDH | | .199 | | 7 | | | .200^*^ | .903 | | 7 | .352 | |
|  | RVH-MDH | | .292 | | 7 | | | .072 | .847 | | 7 | .116 | |
|  | RVH-HDH | | .237 | | 7 | | | .200^*^ | .830 | | 7 | .080 | |
| ERK | Control | | .258 | | 7 | | | .174 | .818 | | 7 | .062 | |
|  | RVH | | .242 | | 7 | | | .200^*^ | .876 | | 7 | .211 | |
|  | RVH-LDH | | .216 | | 7 | | | .200^*^ | .937 | | 7 | .614 | |
|  | RVH-MDH | | .298 | | 7 | | | .061 | .846 | | 7 | .113 | |
|  | RVH-HDH | | .203 | | 7 | | | .200^*^ | .942 | | 7 | .659 | |
| Cyclophilin | Control | | .296 | | 7 | | | .063 | .840 | | 7 | .099 | |
|  | RVH | | .223 | | 7 | | | .200^*^ | .920 | | 7 | .468 | |
|  | RVH-LDH | | .195 | | 7 | | | .200^*^ | .895 | | 7 | .302 | |
|  | RVH-MDH | | .154 | | 7 | | | .200^*^ | .955 | | 7 | .773 | |
|  | RVH-HDH | | .208 | | 7 | | | .200^*^ | .906 | | 7 | .371 | |
| *. This is a lower bound of the true significance. | | | | | | | | | | | | |  |
| a. Lilliefors Significance Correction | | | | | | | | | | | | |  |
