# Supplementary figures and images for: Hibiscus attenuates renovascular hypertension–induced aortic remodeling dose dependently: the oxidative stress role and Ang II/cyclophilin A/ERK1/2 signaling
Source: Front Physiol. 2023 Jun 21;14:1116705. doi: 10.3389/fphys.2023.1116705 (PMC10321301; doi:10.3389/fphys.2023.1116705)

## Supplementary Figure 1

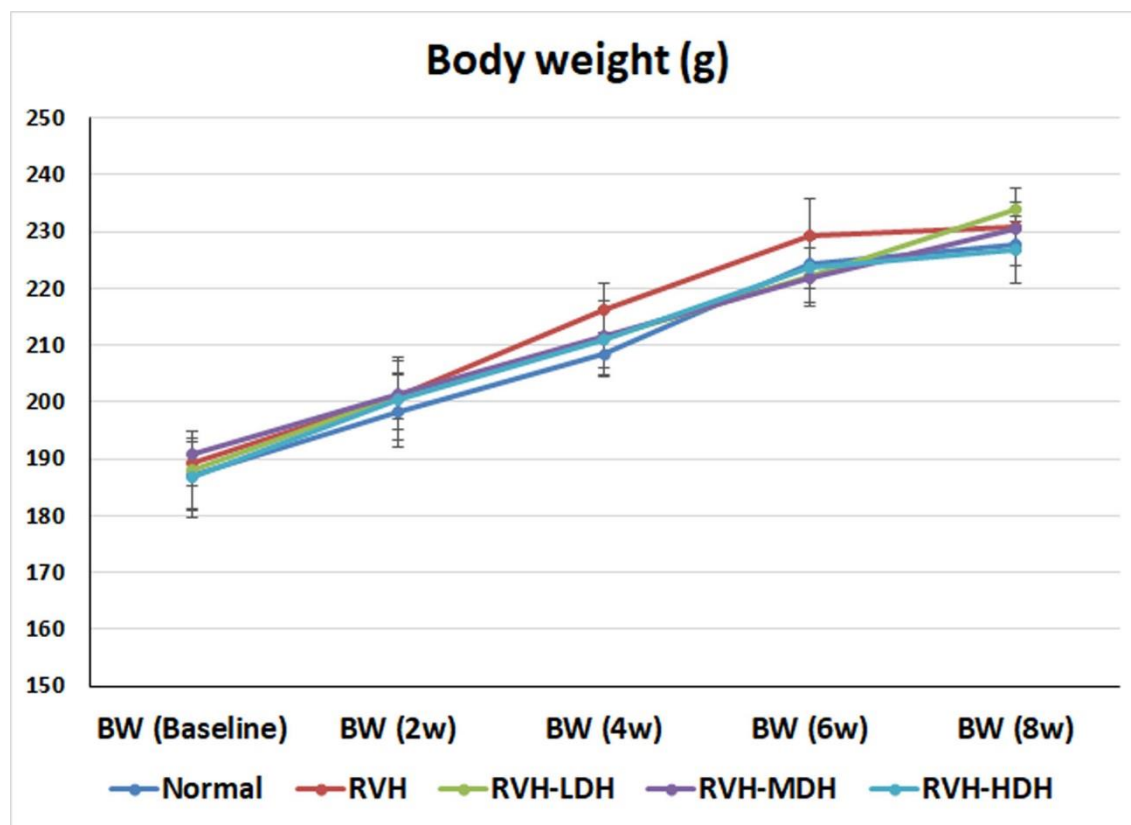

## Supplementary Figure 2

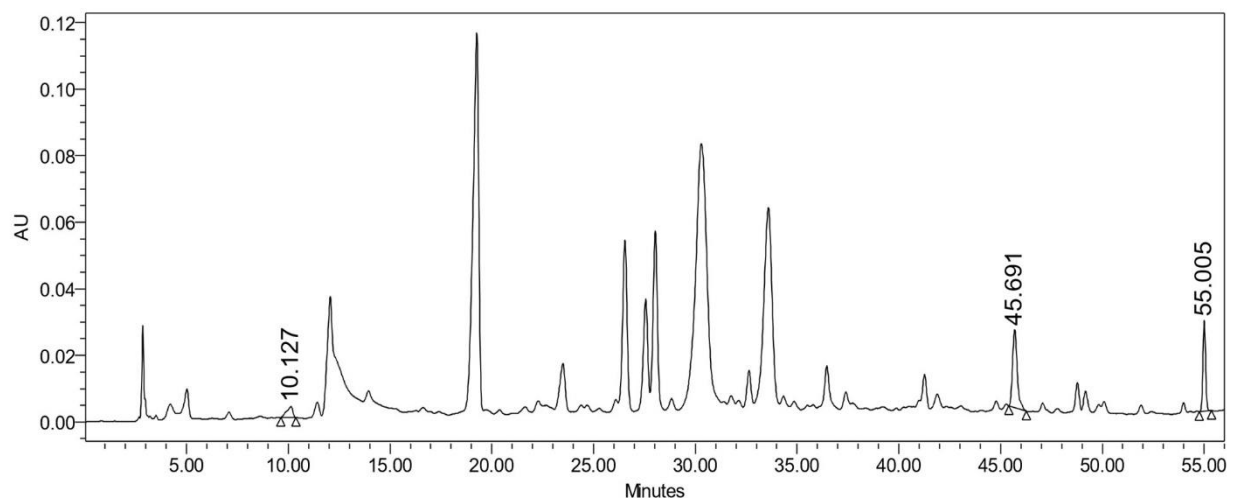

Supplement: Supplementary file 3 [file DataSheet1.pdf]
